# The Farnesyltransferase β-Subunit Ram1 Regulates *Sporisorium scitamineum* Mating, Pathogenicity and Cell Wall Integrity

**DOI:** 10.3389/fmicb.2019.00976

**Published:** 2019-05-08

**Authors:** Shuquan Sun, Yizhen Deng, Enping Cai, Meixin Yan, Lingyu Li, Baoshan Chen, Changqing Chang, Zide Jiang

**Affiliations:** ^1^Guangdong Province Key Laboratory of Microbial Signals and Disease Control, College of Agriculture, South China Agricultural University, Guangzhou, China; ^2^Sugarcane Research Institute, Guangxi Academy of Agricultural Sciences, Nanning, China; ^3^State Key Laboratory of Conservation and Utilization of Subtropical Agro-Bioresources, Guangxi University, Nanning, China

**Keywords:** cell wall integrity, mating, *RAM1*, Ras, *Sporisorium scitamineum*

## Abstract

The basidiomycetous fungus *Sporisorium scitamineum* causes a serious sugarcane smut disease in major sugarcane growing areas. Sexual mating is essential for infection to the host; however, its underlying molecular mechanism has not been fully studied. In this study, we identified a conserved farnesyltransferase (FTase) β subunit Ram1 in *S. scitamineum*. The *ram1*Δ mutant displayed significantly reduced mating/filamentation, thus of weak pathogenicity to the host cane. The *ram1*Δ mutant sporidia showed more tolerant toward cell wall stressor Congo red compared to that of the wild-type. Transcriptional profiling showed that Congo red treatment resulted in notable up-regulation of the core genes involving in cell wall integrity pathway in *ram1*Δ sporidia compared with that of WT, indicating that Ram1 may be involved in cell wall integrity regulation. In yeast the heterodimeric FTase is responsible for post-translational modification of Ras (small G protein) and a-factor (pheromone). We also identified and characterized two conserved Ras proteins, Ras1 and Ras2, respectively, and a *MAT-1* pheromone precursor Mfa1. The *ras1*Δ, *ras2*Δ and *mfa1*Δ mutants all displayed reduced mating/filamentation similar as the *ram1*Δ mutant. However, both *ras1*Δ and *ras2*Δ mutants were hypersensitive to Congo red while the *mfa1*Δ mutant was the same as wild-type. Overall our study displayed that *RAM1* plays an essential role in *S. scitamineum* mating/filamentation, pathogenicity, and cell wall stability.

## Introduction

Sugarcane smut disease caused by the basidiomycetous fungus *Sporisorium scitamineum* is one of the most severe diseases in world-wide sugarcane growing areas. The three-stage morphological transitions are most prominent during the smut fungus life cycle: the haploid sporidia switch to dikaryotic hyphal growth to infect the plant cane; in the later growth period, diploid teliospores form within the host stem; and after teliospore germination, haploid sporidia are generated by successive budding (Taniguti et al., 2015). Therefore switching from non-pathogenic yeast-like sporidia to pathogenic hypha through sexual mating between two compatible sporidia is a prerequisite for infection. In the phytopathogenic basidiomycete model fungus, *Ustilago maydis*, biallelic a locus and multiallelic b locus comprise the tetrapolar mating system. The a locus encodes pheromone precursor *MFA1/2* and receptor *PRA1/2*, controlling sporidial recognition and fusion during the sexual mating process. The b locus encodes bE and bW proteins forming a heterodimeric transcription factor, activating the filamentation (Hartmann et al., 1996; Kahmann and Kamper, 2004). In *S. scitamineum* mfa2 knock-out mutants in *MAT-2* background were mating-deficient (Lu et al., 2017), and b locus deletion mutants were defective in filamentous growth (Yan et al., 2016a), suggesting common molecular characters between *S. scitamineum* and *U. maydis*. However, the molecular mechanisms of *S. scitamineum* mating/filamentation and/or pathogenicity remain largely unclear.

The biogenesis of mature pheromones has been well elaborated in *Saccharomyces cerevisiae* for its pheromone a-factor (Michaelis and Barrowman, 2012), during which the a-factor precursor undergoes a sequential processing by three separate modules, namely the C-terminal processing (prenylation by farnesyltransferase Ram1/Ram2, proteolysis by endoproteases Ste24 or Rce1, and Carboxymethylation by methyltransferase Ste14), the N-terminal proteolytic cleavage by endoproteases Ste24 and Axl1, and non-classical export by the ATP binding cassette (ABC) transporter Ste6 (Young et al., 2005; Paumi et al., 2009). The prenylation of the secreted a-factor-like pheromones is a common feature of ascomycetes and basidiomycetes (Brown and Casselton, 2001; Whiteway, 2011), including *U. maydis* (Spellig et al., 1994; Koppitz et al., 1996), *Ustilago hordei* (Kosted et al., 2000), *Cryptococcus neoformans* (Shen et al., 2002), *Schizophyllum commune* (Fowler et al., 1999), and *Sporisorium reilianum* (Schirawski et al., 2005).

Prenylation is a post-translational modification in which hydrophobic groups are added to the C-terminus of the protein (Michaelis and Barrowman, 2012). Modification of proteins at C-terminal cysteine residue(s) by the isoprenoids farnesyl (C15) and geranylgeranyl (C20) as lipid donors is essential for the biological function of a number of eukaryotic proteins (Leach and Brown, 2012). Three highly conserved prenyltransferases have been described in eukaryotic cells, and are responsible for these protein modifications: protein farnesyltransferase (FTase) and protein geranyltransferase (GGTase) types I and II. β subunit Ram1 (Ras and a-factor maturation) and α subunit Ram2 comprising the heterodimeric FTase (Omer and Gibbs, 1994). Ultimately, yeast and mammalian Ras proteins, yeast a-factor, and the mammalian nuclear scaffold protein lamin B, share a common motif CAAX (“C” is cysteine, “A” is often an aliphatic amino acid, and “X” is any residue except Phe or Leu) at their C-terminus, which are farnesylated by Ram1/Ram2 (Leung et al., 2006). The biological functions of FTase have been investigated by characterizing *RAM1*-deletion mutants in *S. cerevisiae* (He et al., 1991), *C. neoformans* (Vallim et al., 2004; Esher et al., 2016), *Candida albicans* (Song and White, 2003) and *Aspergillus fumigatus* (Qiao et al., 2017), while the *RAM2* gene seems to be essential. However, the *RAM1* function has not been reported in *S. scitamineum*.

In this study, we identified and characterized conserved *RAM1*, *RAS1*, *RAS2*, and *MFA1* genes in *S. scitamineum*. The *ram1*Δ, *ras1*Δ, *ras2*Δ, and *mfa1*Δ mutants all displayed reduced mating/filamentation compared with wild-type, which could be differentially restored by exogenous addition of Synthetic (farnesylated) Mfa1 peptide or cAMP. The *ram1*Δ mutant displayed enhanced tolerance, while the *ras1*Δ and *ras2*Δ mutants were hypersensitive toward cell wall stressor Congo red. However, and the *mfa1*Δ mutant was comparable with WT in stress tolerance to Congo red. In summary, we here report that the FTase β subunit Ram1 as a critical virulence factor in sugarcane smut fungus *S. scitamineum*. Ram1 and Ras proteins are both involved in cell wall integrity pathway of *S. scitamineum* but maybe of different signaling pathway.

## Materials and Methods

### Multiple Sequence Alignment and Phylogenetic Analysis

Amino acid sequences were aligned by ClustalX 2.0 (Thompson et al., 1994) with the following parameters: pairwise alignment parameters (gap opening = 10, gap extension = 0.1) and multiple alignment parameters (gap opening = 10, gap extension = 0.2, transition weight = 0.5, delay divergent sequences = 25%). The alignment was phylogenetically analyzed with maximum likelihood by MEGA 6.0 (Kenaley et al., 2018), using a Le-Gasquel amino acid replacement matrix with 1,000 bootstrap replications. The tree graph was viewed in Tree-View1.1. The alignment of amino acid sequence was adjusted in BioEdit software.

### Strains and Growth Conditions

Two *S. scitamineum* wild-type haploid *MAT-1* (a1b1) and *MAT-2* (a2b2) isolates were obtained from teliospores on sugarcane in Guangdong providence, China (Yan et al., 2016c), and stored locally. The culture medium used in this study included YePSA medium (yeast extract 1%, peptone 2%, sucrose 2%, and agar 2%) and YePSL medium (yeast extraction 1%, peptone 2%, sucrose 2%). For mating and stress tolerance assays, *S. scitamineum* strains were grown in shake culture in YePSL at 28°C for 12 h. The sporidia were harvested via centrifugation and washed twice in distilled water, and concentrations adjusted to obtain OD600 = 1.0 for serial 10-fold dilutions in distilled water (Krombach et al., 2018). The diluted sporidial suspensions of compatible mating type were mixed together in equal volume and plated on YePSA, incubated in dark at 28°C for 48 h before assessment, and photographed. For stress tolerance assessment, the sporidial culture at OD600 = 1.0 and its serial 10-fold dilutions were spotted on MM medium (Chakraborty et al., 1991) in the absence or presence of stress inducers, including 20 μg/mL Congo red (GENVIEW, DC241), 25 μg/mL calcofluor white (Sigma, 18909), 20 μg/mL SDS (GENVIEW, GS286), 1 M Sorbitol (TGI, S0065), 0.5 mM H_2_O_2_ (Damao, 7722841), and incubated in dark at 28°C for 48 h before assessment and photographing. For testing the effects of exogenous cyclic AMP (cAMP; Sigma, A9501) was added to MM plates to reach final concentrations of 2.5 or 5 mM. For testing the effect of *MAT-1* pheromone, exogenous synthetic Mfa1 (Sigma, with or without farnesylation at C-terminal CAAX motif) was added to MM to reach a final concentration of 1 or 10 μg/mL. For colony counting, the *S. scitamineum* strains were shake cultured in YePSL at 28°C for 12 h, before harvesting. Sporidia were washed twice and diluted in distilled water to OD600 = 1.0, from which serial 10-fold dilutions were generated in distilled water to an OD600 of 10^–5^. An aliquot of 150 μL of this diluted sporidial suspension was incubated on solid medium at 28°C, and the colony forming unit (CFU) was counted after 48 h. At least three independent biological replicates of colony counting were performed each time.

### Strains for Gene Deletion and Complementation

Deletion of targeted genes (listed in Supplementary Table S1) follows the same strategy previously described (Li et al., 2014). For generation of deletion mutants, the 1–1.5 kb left and 1–1.5 kb right borders of each targeted gene were PCR-amplified from *S. scitamineum* wild-type genomic DNA separately, and two overlapping *HPT* fragments (for hygromycin resistance) were amplified from pDAN (HYG^R^). These PCR products were used as templates in fusion PCR to generate two PCR fragments separately containing the left or right border of targeted genes with the truncated, partial-overlapped *HPT* fragments. These two fusion-PCR products were transformed into *S. scitamineum* wild-type *MAT-1* protoplasts via polyethylene glycol (PEG)-mediated transformation (Deng et al., 2018).

For *RAM1*, *RAS1*, *RAS2*, and *MFA1* complementation, a fragment containing the 1–1.5 kb 5′ flanking sequence, whole coding sequence and 1 kb 3′ flanking sequence of the respective gene was PCR amplified with wild-type genomic DNA as template, using the primers pair listed in Supplementary Table S2. This fragment was inserted into the engineered pEX2 (Zeocin^R^) derived plasmid (Williams and Birnbaum, 1988) through homologous recombination by using ClonExpress II One Step Cloning Kit (Vazyme, C112-01), to form the complementary plasmid pEX2-RAM1, pEX2-RAS1, or pEX2-RAS2, respectively. The complementary plasmid was, respectively, transformed into the corresponding deletion mutant via PEG-mediated transformation and screened by Zeocin resistance, and confirmed by PCR and Southern blot analysis.

For construction of *eGFP*-*RAM1* plasmid, coding sequence of eGFP was PCR amplified using pEX1 (Sun et al., 2014) as template, and 1818 bp of *RAM1* coding sequence was PCR amplified using genomic DNA of wild-type strain as template. These two PCR amplified fragments were then fused by PCR amplification and cloned into the engineered pEX2 (Zeocin^R^) derived plasmid, driven by a constitutive (*G3PD*) promoter. The two fusion homologous fragments were PCR amplified as one fragment containing HPT-LB fused with complete *G3PD*-*eGFP*-*RAM1* sequence and partially overlapped fragments of the zeocin gene, and the other containing partially overlapped fragments of the zeocin gene and HPT-RB. This construct was used to replace the hygromycin gene (*HPT*) in the *ram1*Δ mutant, with complementary *eGFP-RAM1* coding sequence and zeocin resistance gene. Primers used in this study are listed in Supplementary Table S2.

### Nucleic Acid Manipulation

Fungal genomic DNA was extracted using a modified SDS method (Yan et al., 2016b). PCR amplification was performed using KOD High-Fidelity DNA Polymerase FX (TOYOBO, KEX-101). Purification of DNA fragments was done using a Gel Extraction Kit (Omega, D2500-02) or a Cycle Pure Kit (Omega, D6492-02). Homologous recombination for fragment ligation with the plasmid was performed using Exnase Multis (Vazyme, C113-01). Total RNA was extracted with Trizol (Invitrogen), and the PrimeScript RT Master Mix (TAKARA, RR036A) was used for cDNA synthesis. NANODROP ONE (Thermo scientific) was used for measuring concentrations and purity testing. In Southern blot assay, restriction enzymes (*HindIII*, *Xba*I, and *Spe*I) used for genomic DNA digestion were from New England Biolabs (United States). For probe preparation, the PCR amplified fragment was purified using Cycle Pure Kit (Omega, D6492-02), and labeled with digoxin using DIG-High Prime DNA Labeling and Detection Starter Kit I (Roche, 11745832910). Probe hybridization was performed using a DIG Probe Synthesis Kit (Roche, 11636090910) and detected by DIG Nucleic Acid Detection Kit (Roche, 11175 041910).

### Plant Infection and Fungal Biomass Assessment

Strains were grown in YePSL medium under shake culture conditions at 28°C for 12 h. The sporidia were harvested via centrifugation and resuspended in distilled water to a final OD600 of 1.0. Sporidial suspension (1 mL) was syringe-injected into the 3-week-old seedlings of the highly susceptible sugarcane cultivar ROC22 (Guangxi Province, China). Mock inoculations were carried out by injecting distilled water. The sugarcane stem tissue used for *S. scitamineum* biomass evaluation was collected at 3 days post inoculation (dpi), and quantification of relative fungal biomass in infected sugarcane stem tissue was performed using the fungal *ACTIN* gene as reference (Brefort et al., 2014). The sugarcane glyceraldehyde dehydrogenase (*GAPDH*) gene served as reference for normalization (Su et al., 2016). The black whip symptoms were evaluated for disease rating (Chang et al., 2018). Three biological repeats, each containing three technical replicas for each sample, were performed.

### Quantitative Real-Time PCR

Quantitative reverse transcription polymerase chain reaction (qRT-PCR) was performed on a qTOWER^3^G (Analytik Jena) using the TB Green Premix Ex Tag (TAKARA, RR820A). The qRT-PCR was run with the following settings: 95°C/3 min – (95°C/10 s – 60°C/30 s –72°C/30 s) × 40 cycles and 72°C 10 s. Relative expression values were calculated with the 2^–ΔΔCt^ method (Livak and Schmittgen, 2001), using *ACTIN* as internal control. Three biological repeats, each containing three technical replicas for each sample, were performed. Primers used in this study are listed in Supplementary Table S3.

### Protoplast Production Assay

For protoplast generation assay the cultured *S. scitamineum* sporidia (OD600 ≈ 0.6) were harvested, and incubated with the lyzing solution (the lyzing enzyme, Sigma L1412, was dissolved in SCS solution comprised of 20 mM trisodium citrate and 1M D-sorbitol, at pH 5.8, to reach a concentration of 15 mg/mL) and incubated for 20 min (Chang et al., 2018). The lysis was stopped by placing the reaction tube on ice. The protoplasts production were examined by microscopy (OLYMPUS, CX21) and counted using a hemacytometer (Jeon et al., 2008). Three biological repeats, each containing three technical replicas for each sample, were performed.

### Malondialdehyde (MDA) Content Measurement

*Sporisorium scitamineum* sporidia (OD600 ≈ 0.1) were harvested, and 150 μL of sporidial suspension was used for culturing on MM plates with or without 25 μg/mL SDS treatment for 48 h at 28°C before determination of MDA content. The sporidia samples were gently scraped from the plates and homogenized in an ice bath with 2.0 mL of 5% trichloroacetic acid solution (m/v), and then centrifuged at 10,000 rpm for 15 min at 4°C. Approximately 2.0 mL of the resulting supernatant was mixed with 1.0 mL of 5% trichloroacetic acid containing 0.67% thiobarbituric acid solution (m/v), and the container heated in boiling water for 5 min. The mixture was rapidly transferred to an ice bath, and measured for its absorbance at 532 nm using a spectrophotometer (METASH, UV5100B). Non-specific turbidity of the sample was corrected by subtracting its absorbance at 600 nm. The MDA concentration was calculated and expressed as μmol/g FW (Lanubile et al., 2015).

### Sporidial Staining and Microscopy

*Sporisorium scitamineum* sporidia (OD600 ≈ 1.0) were harvested via centrifugation and washed twice, before further processing and staining. The sporidial suspension with or without SDS in full 200 μg/mL SDS treatment for 10 min was harvested and washed twice in distilled water, and subjected to staining with propidium iodide PI (Sigma, 25535-16-4), by incubating with 60 μg/mL PI solution (in DPBS, Dulbecco’s Phosphate-Buffered Saline: 2.67 mM KCl, 1.47 mM KH_2_PO_4_, 138.00 mM NaCl, and 8.10 mM Na_2_HPO_4_) for 15 min in the dark (Silva et al., 2005). The stained sporidia were washed twice and placed on a slide for examination under an epifluorescent microscope (OLYMPUS, BX53) using a DAPI filter. For Concanavalin A (ConA) staining, 100 μL of sporidial suspension was collected and stained in 100 μg/mL ConA type VI conjugated to FITC (Sigma, C7642) for 45 min as described (Li et al., 2014). The stained sporidia were washed twice and observed by epifluorescent microscopy using a GFP filter. For staining with the fluorescent brightener calcofluor white (CFW), 100 μL of sporidial suspension were collected and stained in 10 μg/mL fluorescent brightener (calcofluor white; Sigma, 18909) as described (Krombach et al., 2018), and examined by epifluorescent microscopy using a DAPI filter. Microscopic images were taken with a digital camera (OLYMPUS, DP80) equipped with the microscope.

### Intercellular cAMP Extraction and Detection

Intracellular cAMP of *S. scitamineum* sporidia were extracted and detected by cAMP Enzyme Immunoassay Kit (Sigma, CA201), using the procedure previous reported (Chang et al., 2018).

## Results

### Identification of a Conserved Farnesyltransferase β Subunit Encoding Gene *RAM1*

**FIGURE 1:**
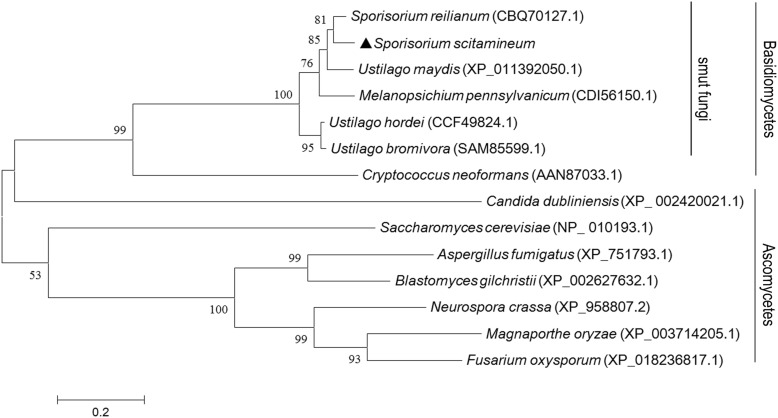
Phylogenetic analysis and amino acid sequences arrangement with *Sporisorium scitamineum Ram1* protein and its orthologs. Phylogenetic analysis of fungal orthologous Ram1 proteins. Amino acid sequences were aligned with ClustalX 2.0 (Thompson et al., 1994) with the following parameters: pairwise alignment parameters (gap opening = 10, gap extension = 0.1) and multiple alignment parameters (gap opening = 10, gap extension = 0.2, transition weight = 0.5, delay divergent sequences = 25%). The alignment was phylogenetically analyzed with maximum likelihood by MEGA 6.0 (Kenaley et al., 2018), by using a Le-Gasquel amino acid replacement matrix with 1,000 bootstrap replications. *S. scitamineum* Ram1 was denoted by a solid triangle.

A tBLASTx search with *Neurospora crassa* Ram1 protein (XM_953714.2:176-1807) as the query revealed the presence of one putative *RAM1* gene in *S. scitamineum.* A phylogenetic analysis of the putative *S. scitamineum* Ram1 protein and previously characterized Ram1 proteins from other fungi, including smut fungi and ascomycetous fungi, indicated the presence of two distinct phylogenetic clades (Figure 1). *S. scitamineum* Ram1 was 87% identical to putative *Sporisorium reilianum* Ram1, 78% to *U. maydis* Ram1, 34% to *C. neoformans* Ram1, and 34% to *A. fumigatus* Ram1, respectively. *RAM1* is predicted to encode a peptide of 606 amino acids, with two prenyltransferase domains and one squalene oxidase repeat (PF00432.16) at positions of 139–180 and 207–250 amino acid in *S. scitamineum*. The domain and amino acid sequences of Ram1 proteins were highly conserved among all smut fungi analyzed (Supplementary Figure S1A).

### *RAM1* Is Required for *S. scitamineum* Mating/Filamentation

**FIGURE 2:**
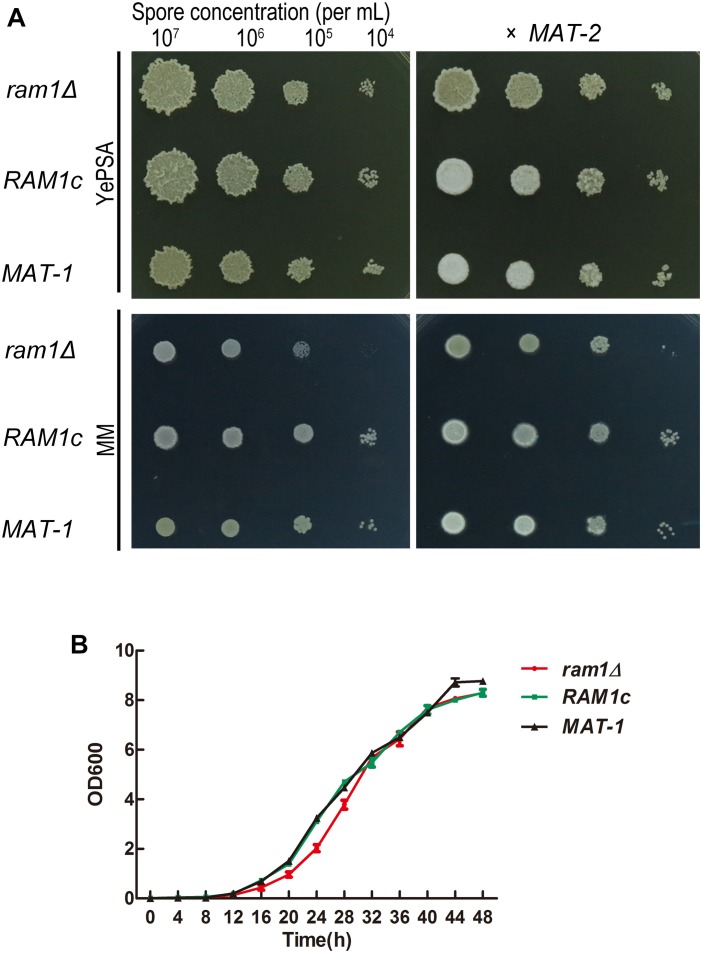
*RAM1* is required for *S. scitamineum* sexual mating. **(A)** Haploid (left) and mating (right, x MAT-2) colony of *ram1*Δ, wild-type *MAT-1* and the complementation strain *RAM1c*. A sporidial suspension (1 μL) of OD600 = 1.0, and the serial 10-fold dilutions of indicated strains, were spotted on YePSL or MM plates and incubated at 28°C for 24 h, before examination. **(B)** Saprophytic growth curve of *ram1*Δ, wild-type *MAT-1* and the complementation strain *RAM1c*. For the growth curve, strains were cultured in YePSL over a period of 48 h. Three biological replicates, each containing three technical replicates for each sample, were performed.

To evaluate the impact of *RAM1* on mating and growth, a *ram1*Δ mutant and its complementation strain *RAM1c* were generated. The number of insertion copy was verified by Southern blotting using *HPT* fragment as probe (Supplementary Figure S1B), and the replacement of *RAM1* gene was also confirmed by Southern blotting using locus-specific probe (Supplementary Figure S1C). The re-introduction of *RAM1* gene was verified by PCR amplification (Supplementary Figure S1D). For haploid vegetative growth, the *ram1*Δ colony surface appeared drier than that of the wild-type *MAT-1* or *RAM1c* strain when grown on YePSA (Figure 2A: left panel), but the growth speed of sporidia was indistinguishable among these three strains when cultured in YePS liquid medium (Figure 2B). The mating of *ram1*Δ sporidia with compatible wild-type *MAT-2* was obviously reduced, compared to that of wild-type or *RAM1c* strains (Figure 2A: right panel). Furthermore, we found that mixing of two *ram1*Δ of compatible mating types displayed a complete blockage of mating and filamentous growth/filamentation (Supplementary Figure S2A). qRT-PCR analysis was performed for assessing expression of genes related to fungal mating and filamentation in mutants and wild-type. The results showed that in *ram1*Δ sporidia, the a locus genes *MFA1* and *PRA1*, and the b locus genes *bE1* and *bW1* were all comparable to that in the complementary or wild-type strains, while *PRF1*, the master transcriptional regulator for mating and filamentation, was significantly down-regulated (Supplementary Figure S2B). Overall these results showed that *Ram1* is required for *S. scitamineum* mating and filamentation.

### Expression of *RAM1* During Development of Sporidia to Hyphal in *S. scitamineum*

**FIGURE 3:**
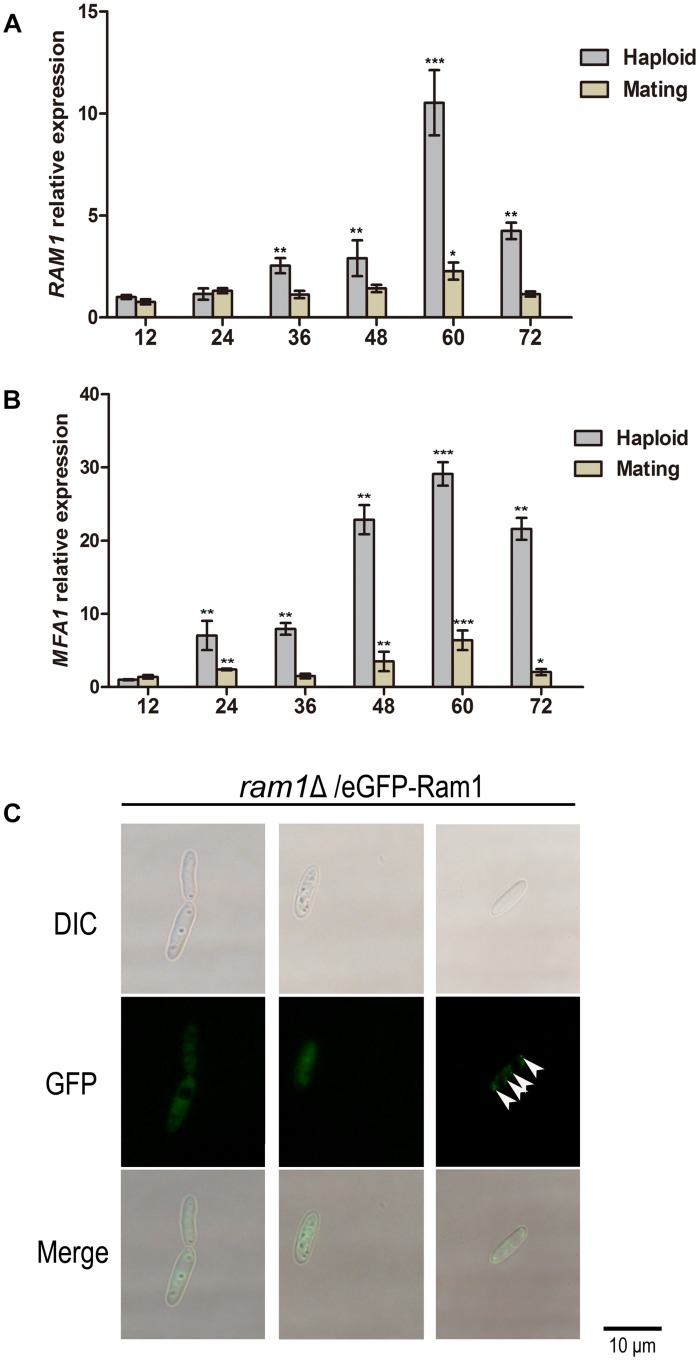
Transcriptional profile of *RAM*1 gene and *MFA1* gene and subcellular localization of Ram1 protein in *S. scitamineum*. **(A)** qRT-PCR analysis of *RAM1* expression in wild-type strain under sporidial growth or mating condition on YePSA plate. *RAM1* expression in 12 h was set as 1 and the relative gene expression fold change in a period of 72 h was calculated with 2^–ΔΔCt^ method (Livak and Schmittgen, 2001), using *ACTIN* as internal control. Statistical significance of the expression data was determined at ^∗∗∗^*p* < 0.005, **p < 0.01, and *p < 0.05 using Student’s *t*-test, and error bars represent ± standard deviation values. **(B)** qRT-PCR analysis of *MFA1* expression in wild-type strain under sporidial growth or mating condition on YePSA plate. *MFA1* expression in 12 h was set as 1 and the relative gene expression fold change in a period of 72 h was calculated with 2^–ΔΔCt^ method (Livak and Schmittgen, 2001), using *ACTIN* as internal control. Statistical significance of the expression data was determined at ^∗∗∗^*p* < 0.005, **p < 0.01, and *p < 0.05 using Student’s *t*-test, and error bars represent ± standard deviation values. **(C)** Localization of eGFP-Ram1 fusion proteins in the *ram1*Δ sporidia. Sporidial suspension of OD600 = 1.0 were spotted on YePSA plates and incubated at 28°C for 24 h, before epifluorescent microscopic observation and imaging. Arrows denote punctate structure visualized by eGFP-Ram1. Scale bar = 10 μm.

To determine the potential function of *RAM1* at the haploid sporidia switch to dikaryotic hyphal growth, we assessed transcriptional profile of *RAM1* gene every 12 h over a period of 72 h in haploid or mating condition using qRT-PCR. In wild-type sporidia, the expression of *RAM1* was elevated with cultural time. The maximal expression level occurred at around 60 h, of more than 10-fold compared to that at 12 h. Then we examined the expression of *RAM1* gene from 12 to 72 h in dikaryotic hyphal stage. The results showed that the expression of *RAM1* was obviously reduced compared to that in sporidia stage (Figure 3A). We also found the pattern of pheromone precursor gene *MFA1* was similar to that of *RAM1* (Figure 3B), but the elevated expression initially started at 48 h and reached the maximum at around 60 h (Figures 3A,B).

To observe the subcellular localization of Ram1 protein, a copy of *eGFP*-*RAM1* fusion fragment was transferred into the *ram1*Δ mutant. The mating ability was restored in the resulting *ram1*Δ/eGFP-Ram1 strain, indicating that the eGFP-Ram1 fusion protein is functional. By epifluorescent microscopy, we observed that the eGFP-Ram1 signal localized in the cytosol of sporidial cells (Figure 3C), with occasional punctate structure in the cytoplasm (Figure 3C, arrows). These data imply that *RAM1* plays an important role in development of sporidia.

### Mfa1 Farnesylation Could Partially Restore *ram1*Δ Mating/Filamentation Defect

**FIGURE 4:**
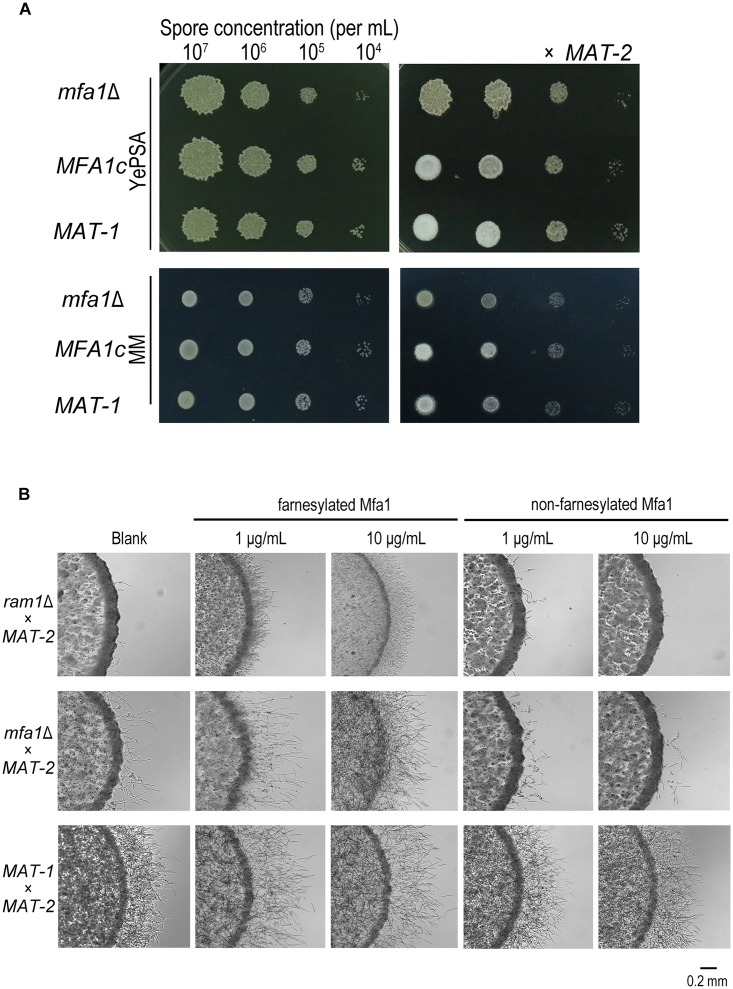
Mfa1 farnesylation could partially restore *ram1*Δ mating/filamentation defect in *S. scitamineum*. **(A)** Haploid (left) and mating (right, x *MAT-2*) colony of *m1*Δ and wild-type *MAT-1*. 1 μL sporidia suspension of OD600 = 1.0, and the serial 10-fold dilutions of indicated strains, were spotted on YePSA or MM plates and incubated at 28°C for 24 h. **(B)** Mating/filamentation assay with synthetic (farnesylated or non-farnesylated) Mfa1 exogenously supplied to MM plates to reach a final concentration of 1 μg/mL or 10 μg/mL. 1 μL sporidial suspension (of the mutants *ram1*Δ, *mfa1*Δ or wild-type strain) of OD600 = 1.0 were, respectively, mixed with compatible mating partner and cultured on MM plates at 28°C for 24 h.

The *S. cerevisiae* and *U. maydis* pheromone precursor Mfa1 undergoes C-terminal farnesylation before its maturation and functioning as a hormone in compatible sporidial recognition and induction of sexual mating (Ashby and Rine, 1995; Koppitz et al., 1996). Therefore we intended to investigate whether the mating/filamentation defect of the *ram1*Δ was due to failure of the Mfa1 precursor in farnesylation (Spellig et al., 1994; Ashby and Rine, 1995). In *S. scitamineum*, the *MFA1* gene codes a 41-amino-acid pheromone precursor in *MAT-1* (CAI59747.1), which contains a predicted farnesylation motif CTIA at its C-terminal. We generated *mfa1*Δ mutants and its complementation strains *MFA1c* with wild-type *MAT-1* background. The mutants and complementation strains were verified by PCR and Southern blotting (Supplementary Figure S3). As expected the mating of the *mfa1*Δ mutant with compatible mating type *MAT-2* was completely blocked when cultured on YePSA media, and significant reduced on MM plates, compared to that of wild-type or *MFA1c* strains (Figure 4A). To confirm that such mating/filamentation defect was caused by loss of functional (farnesylated) a-pheromone encoded by *MFA1*, we generated and supplied the synthetic Mfa1 peptide with or without C-terminal farnesylation to MM plates to reach a final concentration of 1 or 10 μg/mL, and tested its effect on restoring mating/filamentation of the *mfa1*Δ or *ram1*Δ mutant. We found that the farnesylated Mfa1 peptide could effectively restore mating/filamentation in the *mfa1*Δ mutant at the concentration of 10 μg/mL, and only partially restored that of the *ram1*Δ mutant (Figure 4B). In contrast, addition of the Mfa1 peptide without farnesylation failed to rescue the defective mating/filamentation in *ram1*Δ or *mfa1*Δ mutant (Figure 4B). Putting together, these results suggested that Ram1 is likely required for farnesylation of pheromone precursor Mfa1, which is essential for *S. scitamineum* mating/filamentation.

### *RAM1* Is Required for Full Pathogenicity of *S. scitamineum*

**FIGURE 5:**
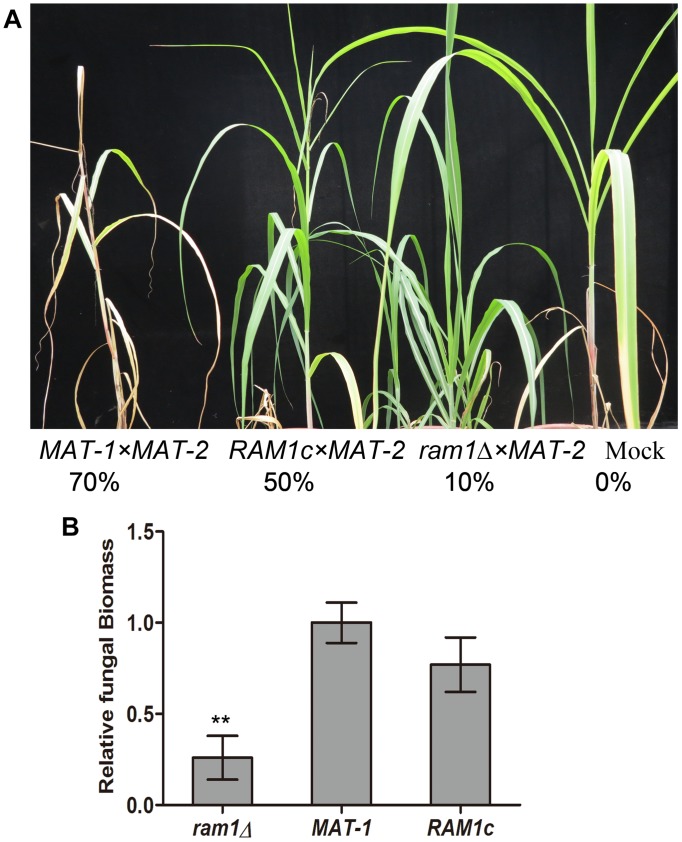
*RAM1* is required for *S. scitamineum* pathogenicity. **(A)** The susceptible sugarcane cultivar ROC22 was inoculated with mixed sporidia (1:1) of the indicated wild-type or mutant combination through injection at the 5–6-leaf seedling stage, and the symptoms of ‘black whip’ were assessed and documented at 200 days post inoculation (dpi). The infection assays were performed with at least 20 seedlings. Percentage of plants with ‘black whip’ symptoms is illustrated. **(B)** Colonization of the plant tissue by *ram1*Δ, wild-type *MAT-1*, or the complementation strain *RAM1c*. Relative fungal biomass was determined by qRT-PCR with *S. scitamineum* genomic DNA isolated from infected sugarcane stems at 3 dpi. The fungal *ACTIN* gene was used for estimation of relative fungal biomass through 2^–ΔΔCt^ method (Livak and Schmittgen, 2001), normalized to the plant *GADPH* gene. Barchart depicts the average ± standard error (SE) derived from three biological replicates. Significance of differences between wild-type *MAT-1* and *ram1*Δ was calculated using Student’s *t*-test, **p < 0.01.

To test whether the *RAM1* is required for *S. scitamineum* pathogenicity, we inoculated the mixture of *ram1*Δ × *MAT-2* sporidia by injection, into a susceptible sugarcane seedling, and evaluated the disease symptoms. Inoculation with the wild-type *MAT-1* × *MAT-2* or the *RAM1c* × *MAT-2* combination served as positive control, and sterile water as mock. The *ram1*Δ × *MAT-2* combination displayed significantly reduced pathogenicity, with only around 10% of the infected seedlings showing black whip symptoms at 200 dpi. In contrast, the wild-type *MAT-1* and *MAT-2* mixture lead to 70% of the infected seedlings with black whip symptoms, while that in the *RAM1c* and wild-type *MAT-2* mixture was approximately 50% (Figure 5A). Consistently, relative fungal biomass was significantly lower at 3 dpi in seedling stems inoculated with *ram1*Δ × *MAT-2*, compared to that inoculated with the wild-type or complementary strain combinations (Figure 5B). These data suggest that *Ram1* is also essential for full pathogenicity of *S. scitamineum*.

### *RAS* Genes Are Required for *S. scitamineum* Mating and Filamentation

It has been reported that Ram1/Ram2 FTase complex controls fungal differentiation and/or pathogenicity via post-translational modifications of Ras proteins, the small G proteins playing an important role in cell signaling (Omer and Gibbs, 1994). Therefore we wondered whether defects mating and filamentation defects in the *ram1*Δ were related to Ras protein modification and the Ras-mediated signaling pathway. A BLASTP search with *U. maydis* Ras1 protein (XP_011386972.1) as the query revealed the presence of one putative *RAS1* gene in *S. scitamineum*, which is predicted to encode a protein of 215 amino acids, with one RAS superfamily domain (PF00071) from amino acids 11–172. A BLASTP search with *U. maydis* Ras2 (XP_011387629.1) as the query revealed the presence of one putative *RAS2* gene in *S. scitamineum*, encoding a protein of 192 amino acids, with one RAS superfamily (PF00071) from amino acids 5–173. *S. scitamineum* Ras1 and Ras2 shared 58% identity from Ras1 amino acids 10–171 covering the entire RAS domain, which indicated the conservation of Ras proteins at the amino acid sequence level. However, this indicated the other portions outside of the RAS domain of the Ras1 and Ras2 proteins probably serve other functions. A phylogenetic analysis of *S. scitamineum* Ras1 and Ras2 proteins with predicted or characterized Ras proteins from other basidiomycetous or ascomycetous fungi indicates that Ras1 and Ras2 proteins are divergent with each other, but is respective well conserved within its own group (Supplementary Figure S4).

**FIGURE 6:**
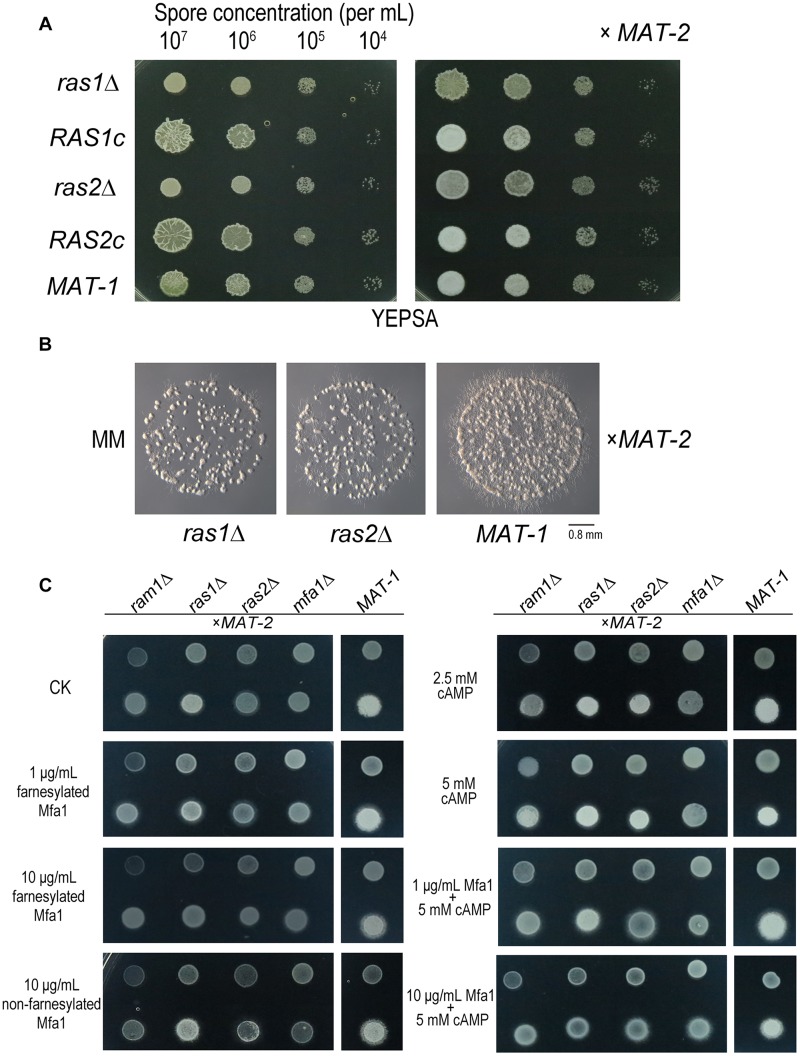
*RAS1* and *RAS2* are required for *S. scitamineum* mating/filamentation. **(A)** Haploid (left) or mating (right, x *MAT-2*) colonies of *ras1*Δ, *RAS1c*, *ras2*Δ, *RAS2c*, and wild-type *MAT-1*. One microliter sporidial suspension of OD600 = 1.0, and serial 10-fold dilutions, of indicated strains, were spotted on YePSA plates and incubated at 28°C for 24 h. **(B)** Microscopic observation of mating colonies of *ras1*Δ, *ras2*Δ, and wild-type *MAT-1* on MM plates with 1 μL sporidial suspension (OD600 = 0.1) and incubated for 12 h at 28°C. Scale bar = 0.8 mm. **(C)** Haploid (upper) and mating (lower, x *MAT-2*) colony of *ras1*Δ, *ras2*Δ, *ram1*Δ, and *mfa1*Δor wild-type *MAT-1* on MM plates with 1 μL OD600 = 1.0 sporidia suspension with exogenous cAMP of 0, 2.5, or 5 mM, synthetic (farnesylated) Mfa1 exogenously supplied to MM plates to reach a final concentration of 1 or 10 μg/mL, synthetic (non-farnesylated) Mfa1 exogenously supplied to MM plates to reach a final concentration of 10 μg/mL at 28°C for 24 h.

To further investigate the function of *S. scitamineum RAS* genes, we generated *ras1*Δ and *ras2*Δ mutants, and their complementation strains *RAS1c*, *RAS2c*, both with wild-type *MAT-1* background, and verified by Southern blotting and PCR (Supplementary Figures S5, S6). The *ras1*Δ haploid colonies were obviously smaller and wetter than that of the wild-type *MAT-1* strain (Figure 6A, left panel). The compatibility of *ras1*Δ with *MAT-2* sporidia was obviously reduced compared to that of wild-type *MAT-1* and the *RAS1* complementation strain, which displayed normal filament formation after mating (Figures 6A right panel, 6B). The *ras2*Δ haploid colonies were also obviously smaller and wetter than that of the wild-type *MAT-1* strain (Figure 6A, left panel). However, the mating of *ras2*Δ with *MAT-2* sporidia was only mildly reduced, and the mating of complementation strains was normal (Figures 6A right panel, 6B). Exogenous applications of cAMP (concentrations from 2.5 to 5 mM) could enhance (but not fully restore) sexual mating of mutants of *ras1*Δ or *ras2*Δ but not the one of *ram1*Δ and *mfa1*Δ (Figure 6C). This result indicates that cAMP signaling may be at downstream of Ras proteins, but not sufficient for fully restore Ram1 or Mfa1 function. Exogenous applications of synthetic (farnesylated) Mfa1 (concentrations 1 or 10 μg/mL) only or together with 5 mM cAMP couldn’t restore sexual mating of mutants of *ram1*Δ, *ras1*Δ, or *ras2*Δ except for *mfa1*Δ, synthetic (non-farnesylated) Mfa1 couldn’t restore mating/filamentation in these four mutants (Figure 6C). The phenotype of mating defect in *ras1*Δ or *ras2*Δ mutants were slighter than that in *ram1*Δ imply function of Ras1/Ras2 proteins are probably redundant in regulating mating/filamentation in *S. scitamineum*.

### Ram1 and Ras Signaling Are Involved in *S. scitamineum* Cell Wall Integrity

Next we evaluated the stress tolerance of the *ram1*Δ, *ras1*Δ, *ras2*Δ, and *mfa1*Δ mutants, toward 20 μg/mL Congo red (cell wall stress), 20 μg/mL SDS (membrane stress), 25 μg/mL CFW (cell wall stress), 1 M Sorbitol (osmotic stress) and 0.5 mM H_2_O_2_ (oxidative stress). The mutants *ram1*Δ, *ras1*Δ, and *ras2*Δ displayed greater growth inhibition by treatment with SDS (20 μg/mL) or H_2_O_2_ (0.5 mM) than the wild-type *MAT-1* strain, and the tolerance abilities were restored in the complementation strains (Supplementary Figures S7–S9). Both *ras1*Δ and *ras2*Δ mutants were hypersensitive to CR (20 μg/mL) while *ram1*Δ mutant was not hypersensitive (Supplementary Figure S7). With respect to osmotic stress sensitivity against sorbitol (1 M), all the mutant strains were indistinguishable from the wild-type strain (Supplementary Figure S7). On the other hand, the *mfa1*Δ mutant displayed no obvious difference in tolerance toward all these testing stressors, compared to that of WT (Supplementary Figure S7).

**FIGURE 7:**
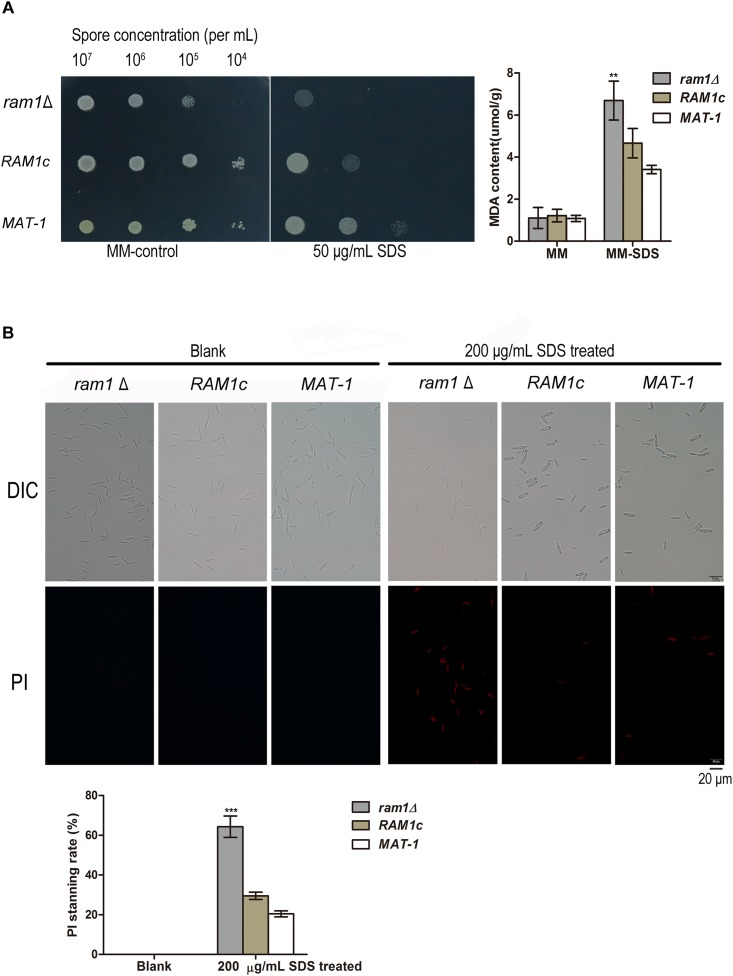
*RAM1* is required for membrane stability of *S. scitamineum* sporidia. **(A)** Malondialdehyde (MDA) content of *ram1*Δ, wild-type *MAT-1* and the complementation strain *RAM1c* with or without treatment of 50 μg/mL SDS. Barchart on the right panel depicts the average ± standard error (SE) derived from three independent biological replicates. Significance of differences between wild-type *MAT-1* and ram1 was calculated by Student’s *t*-test, **p < 0.01. **(B)** Microscopic examination (upper panel) of *ram1*Δ, wild-type *MAT-1* and the complementation strain *RAM1c* with Propidium iodide (PI) staining after 200 μg/mL SDS treatment for 10 min. PI staining was assessed after 15 min of staining. Scale bar = 20 mm. Barchart on the lower panel depicts the average ± standard error (SE) derived from three independent replicates. Significance of differences between wild-type *MAT-1* and *ram1*Δ was calculated using a Student’s *t*-test, ***p < 0.005.

Given that the *ram1*Δ mutant was hypersensitive toward SDS, we intended to measure malondialdehyde (MDA) content of *S. scitamineum* sporidia, which indicates the degree of cell membrane damage (Li et al., 1998). The results showed that the *ram1*Δ sporidia contained notably higher MDA content than that of the wild-type *MAT-1* or the complementation strain after SDS treatment (Figure 7A), which may account for reduced tolerance to SDS in the *ram1*Δ. It has been reported that SDS strongly increases solubilization of membrane lipid, leading to lipid peroxidation and cell membrane damage (He et al., 2005). MDA is the final product, and also an important indicator, of lipid oxidation (Lanubile et al., 2015), hence it is expected that the increased MDA content indicates cell membrane damage caused by SDS treatment. Correspondingly, the *ram1*Δ sporidia displayed notable propidium iodide (PI) positive staining compared to that of the wild-type or the complementation strains, after treatment with 200 μg/mL SDS for 10 min (Figure 7B), suggesting that the cell membrane of *ram1*Δ sporidia was weaker than that of the wild-type or the complementation strains.

**FIGURE 8:**
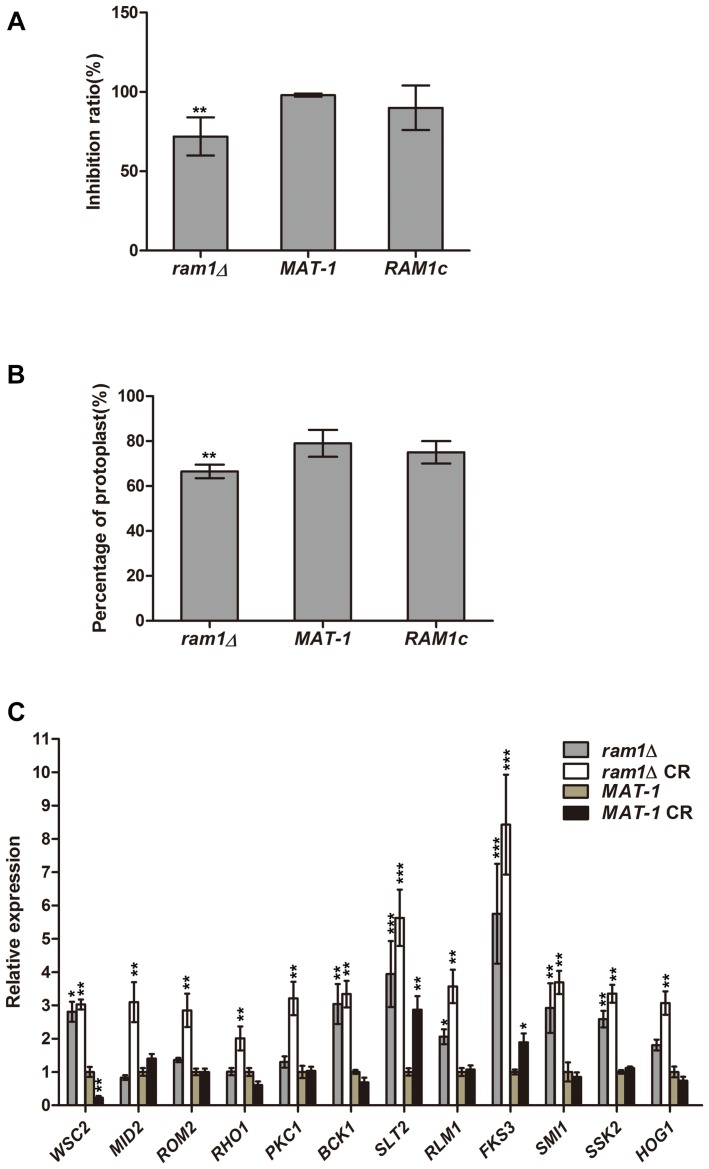
*RAM1* regulates *S. scitamineum* cell wall integrity. **(A)** Growth inhibition calculated by number of colonies formed following streaking of *ram1*Δ, wild-type *MAT-1* and the complementation strain *RAM1c* sporidial suspension (OD = 10^–5^; 150 mL), on MM plates with or without 50 μg/mL Congo red (CR). Statistical significance of the inhibition was determined at **p < 0.01 using Student’s *t*-test, and mean ± standard deviation was derived from three independent biological replicates. **(B)** Enzymatic digestion and protoplast production assay with *ram1*Δ, wild-type *MAT-1* and the complementation strain *RAM1c*. Protoplast production was counted at 20 min post lyzing enzyme incubation. Statistical significance of the protoplast production was determined at **p < 0.01 using Student’s *t*-test, and mean ± standard deviation was derived from three independent biological replicates. **(C)** qRT-PCR analysis with genes involved in cell wall integrity pathway in *ram1*Δ and wild-type *MAT-1*, with or without treatment of 25 μg/mL CR. Gene expression in the wild-type *MAT-1* strain (untreated) was set as 1 and the relative gene expression fold change in other strains/conditions was calculated with 2^–ΔΔCt^ method (Livak and Schmittgen, 2001), using *ACTIN* as internal control. Barchart depicts the average ± standard error (SE) derived from three independent biological replicates. Statistical significance of the expression data was determined at ***p < 0.005, **p < 0.01, *p < 0.05 using Student’s *t*-test, and error bars represent ± standard deviation values.

On MM media supplemented with 20 μg/mL Congo red (CR), the *ram1*Δ mutant displayed no obvious difference in growth compared to the wild-type strain (Supplementary Figure S7). We further verified the inhibition effect of CR by colony counting from the sporidial suspension on the MM plates with or without 50 μg/mL CR. Growth inhibition of *ram1*Δ mutant on CR-supplemented MM plate was 71.94% while that of wild-type or the complementation strain was 98%, of that on the MM plate without CR (Figure 8A). This indicates that the *ram1*Δ mutant was more tolerant toward CR compared to the wild-type or the complementation strains. To test cell wall integrity, an equal amount of sporidia from the *ram1*Δ mutant, the *RAM1c* and wild-type *MAT-1* strains were treated with 15 mg/mL cell wall lyzing enzyme at 28°C for 20 min. The percentage of protoplasts generated was viewed as an indicator of cell wall integrity. The result showed that the *RAM1c* and wild-type *MAT-1* sporidia were hypersensitive to the enzyme treatment, producing more protoplasts than the *ram1*Δ mutant (Figure 8B). This also suggested that the cell wall integrity was enhanced in the *ram1*Δ mutant, and thus it was more tolerant to CR. Furthermore, we assessed the expression of the genes involved in the cell wall integrity (CWI) pathway by qRT-PCR, in the *ram1*Δ mutant in comparison of the wild-type *MAT-1*, with or without treatment of 25 μg/mL CR. Our results showed that *MID2* and *WSC1* (membrane proteins as main sensors) (Philip and Levin, 2001), *ROM2* (GEF which sensors interacted with) (Ozaki et al., 1996), *RHO1* (small GTPase) (Schmelzle et al., 2002), *PKC1* (protein kinase c) (Paravicini and Friedli, 1996), *BCK1* (MAPKKK) (Lee and Levin, 1992), *MKK1* (MAPKK) (Kim et al., 2008), *SLT2* (MAPK) (Rui and Hahn, 2007), *RLM1* (downstream transcription factors) (Jung et al., 2002), *FKS3* and *SMI1* (glycan synthase) (Lesage and Bussey, 2006) were all significantly up-regulated in the *ram1*Δ mutant treated with CR, compared to wild-type *MAT-1* sporidia (Figure 8C). This result indicated that the *RAM1* gene may regulate CWI via transcriptional regulation of the genes involved in this signaling pathway.

**FIGURE 9:**
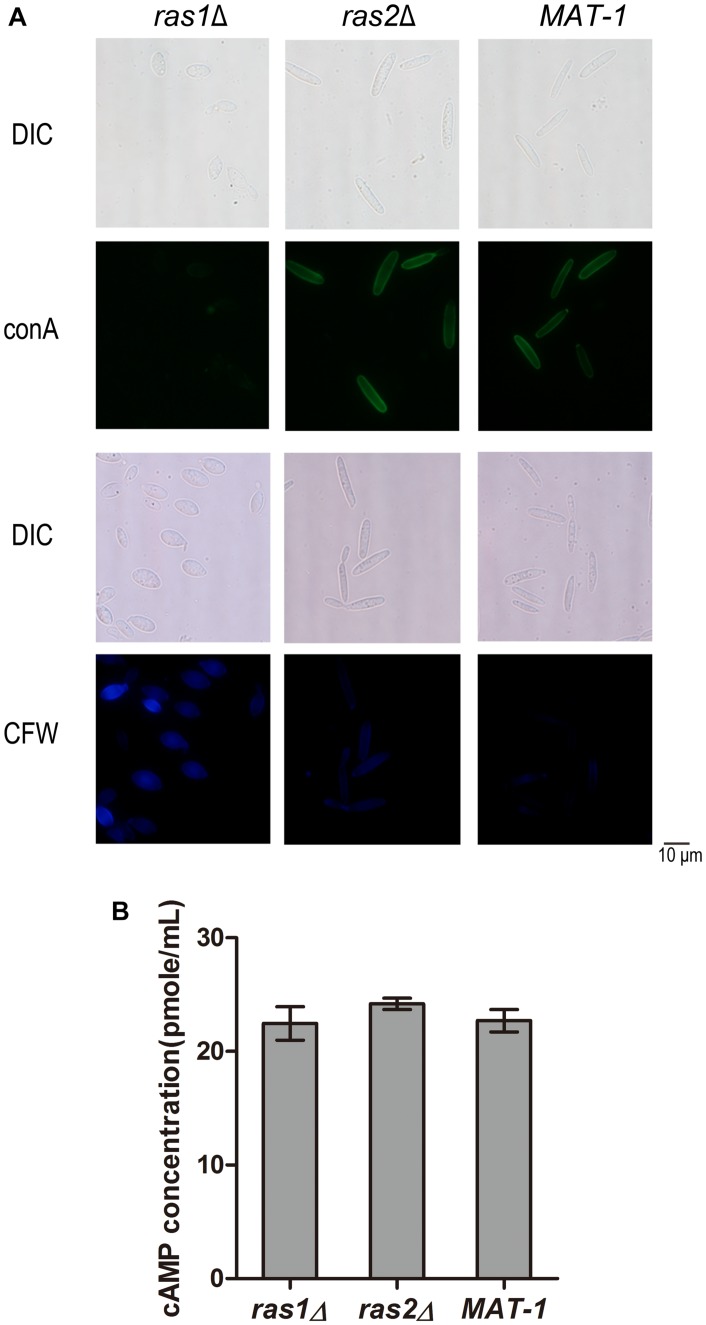
*RAS1* and *RAS2* regulates *S. scitamineum* cell wall integrity. **(A)** Microscopic examination of *ras1*Δ, *ras2*Δ, and wild-type *MAT-1* stained with 100 μg/mL Concanavalin A (conA, type VI conjugated to FITC), or with 10 μg/mL fluorescent brightener calcofluor white (CFW). Scale bar = 10 mm. **(B)** Measurement of intracellular cAMP in 2-day-old sporidia of strains *ras1*Δ, *ras2*Δ and wild-type *MAT-1*, cultured on YePSA plates at 28°C for 24 h. The cAMP concentration was calculated and expressed as pmol/mL. Barchart depicts the average ± standard error (SE) derived from three independent replicates. Statistical analysis was performed by Student’s *t*-test.

On the other hand, the *ras1*Δ and *ras2*Δ mutants displayed hypersensitivity to cell wall stressors CR, CFW, and SDS, and the tolerance abilities were restored in the complementation strains (Supplementary Figures S7, S9). We further investigated whether *Ras1* and/or *Ras2* affect the cell wall integrity by conA and CFW staining. ConA is lectin specifically binding to internal and non-reducing terminal α-d-mannosyl groups and thus is routinely used to assay the presence of mannan moieties in fungal cell walls (Maddi et al., 2012). Our results showed that the *ras1*Δ mutant exhibited weaker fluorescence staining by conA, indicating reduced conA binding sites, while the *ras2*Δ mutant displayed indistinguishable conA staining compared to that of the wild-type (Figure 9A). For CFW staining, the *ras1*Δ mutant exhibited a significantly brighter staining pattern than the wild-type, and the *ras2*Δ mutant was slightly brighter than wild-type *MAT-1* (Figure 9A). This result implied that the cell wall permeability toward CFW dye was enhanced in the *ras1*Δ and the *ras2*Δ mutants, but not in wild-type.

In *U. maydis*, both Ras1 and Ras2 regulate intercellular cAMP level (Nadal et al., 2008; Zhu et al., 2009). In *C. neoformans*, intercellular cAMP level regulates the cell wall integrity pathway (Donlin et al., 2014). To furthermore evaluate the role of cAMP in cell wall integrity we measured the intercellular cAMP concentration in the *ras1*Δ and *ras2*Δ mutants in comparison with wild-type. The results showed no obvious differences in the intercellular cAMP concentrations between the *ras1*Δ and the *ras2*Δ mutants and the wild-type strain (Figure 9B). We proposed that *Ras1*/*Ras2*-regulating CWI pathway is likely independent of cAMP/PKA signaling pathway.

Overall, in this section we found that Ram1 may negatively, and Ras proteins positively, regulate CWI pathway in *S. scitamineu*m. The a-factor Mfa1 may not be involved in *S. scitamineum* CWI regulation.

## Discussion

The predominant function of FTase composed of Ram1 and Ram2 is conserved among eukaryotes (Omer and Gibbs, 1994). In our study, the *ram1*Δ mutant is viable, whereas the *ram2*Δ mutants could not be generated although repeated attempts were made, which is consistent with what has been reported in *S. cerevisiae, C. neoformans*, and *C. albicans* that the *RAM2* gene is essential while *RAM1* is not (Alspaugh et al., 2000; Song and White, 2003; He et al., 2005). It remains a possibility that some suppressors can directly or indirectly enhance cross-prenylation of certain CAAX substrates to replace the function of Ram1 but not Ram2. *S. cerevisiae* Ram1 is associated to sexual mating and survival in stressful conditions including high temperature, but is not essential for haploid growth (Michaelis and Barrowman, 2012). However, *C. neoformans* Ram1 is involved in diploid growth (Nadal et al., 2008). In *A. fumigatus*, *ramA*Δ mutants resulted in growth abnormalities including impaired hyphal branching, delayed conidial germination, reduced conidial viability, and aberrant distribution of nuclei in growing hyphae (Fortwendel et al., 2004). In this study, we found that the virulence of the *S. scitamineum ram1*Δ mutant (mixed with compatible wild-type sporidia) was significantly reduced compared to the wild-type strain, possibly resulting from reduced mating/filamentation and/or weakening stress resistance. For the Ram1 subcellular localization, we generated an eGFP-Ram1 strain and found it localized in the cytosol of sporidial cells. We made attempts to use the native promoter fused with eGFP-Ram1, but we found such ectopic expression of *RAM1* was less than 20% compared to wild-type. Relative expression of *RAM1* driven by G3PD promoter in *ram1*Δ/eGFP-Ram1 strain was about 70% compared to that of wild-type and the mating ability was restored (data not shown). Therefore, we believe in the localization of Ram1 protein was original not GFP-artifactual. *S. scitamineum ram1*Δ mutant showed notably down-regulation of the *PRF1* gene, encoding a master transcription factor governing mating and filamentation, which may account for its defect in mating/filamentation. However, we did not find down-regulation of the a or b locus genes, which were shown to be transcriptionally induced by Prf1 in *U. maydi*s mating/filamentation (Hartmann et al., 1999). We infer that *S. scitamineum* may possess redundant (un-identified) regulator of the a or b locus genes’ transcription, which may not depend on Ram1 function.

In *S. cerevisiae*, a-factor precursor (with CVIA C-terminal motif), Ras1 (with CIIC C-terminal motif), Ras2 (with CIIS C-terminal motif), G protein gamma subunit Ste18 (with CTLM C-terminal motif) are farnesylated by the FTase composed of Ram1 and Ram2 (Omer and Gibbs, 1994; Flom et al., 2008; Michaelis and Barrowman, 2012). Currently, fungal Ras1 and Ras2 are known to be involved in regulating not only growth, development and conidiation, but also virulence and tolerance to oxidation, cell wall disturbance, or heat sensitivity in either independent or interactive manners (Mosch et al., 1996; Lee and Kronstad, 2002; Bluhm et al., 2007; Xie et al., 2013). Disruption of fungal Ras2 has been previously reported in several fungi and also in this study, but deletion of *RAS1* gene was only reported in haploid pathogenic fungus *A. fumigatus* (Fortwendel et al., 2004; Boyce et al., 2005). It was reported that in *U. maydis* or *C. albicans*, both Ras1 and Ras2 regulate intracellular cAMP level, with Ras1 as cAMP-dependent, and Ras2 as MAPK-dependent (Nadal et al., 2008; Zhu et al., 2009). In this study, we successfully deleted *RAS1* gene in *S. scitamineum*. Both *S. scitamineum ras1*Δ and *ras2*Δ mutants showed defective sexual mating which could enhance but not restore obviously by exogenous cAMP. To evaluate whether the concentration of intercellular cAMP of *ras1*Δ and *ras2*Δ mutants changed, ELISA method was conducted and the results showed no obvious differences in the intercellular cAMP concentrations between the *ras1*Δ and the *ras2*Δ mutants and the wild-type strain. Similarly, in *S. pombe* and *Schizophyllum commune*, deletion of *RAS1* couldn’t reduce the cAMP; in *Fusarium graminearum*, the intercellular cAMP level was not affected by deletion of *RAS2* (Hughes et al., 1993; Yamagishi et al., 2004; Bluhm et al., 2007). On the other hand, we found that exogenous addition of synthetic (farnesylated) Mfa1 peptide could effectively restore mating/filamentation in the *S. scitamineum mfa1*Δ mutant, and partially restore that in the *ram1*Δ *ras1*Δ and *ras2*Δ mutants; synthetic (non-farnesylated) Mfa1 couldn’t

restore mating/filamentation in these four mutants. We infer that both Mfa1 farnesylation and Ras-mediated signaling pathway are critical for *S. scitamineum* mating/filamentation, and may both depend on Ram1 function. It would be worth generating and assessing mislocalization of Ras and Mfa1 proteins by comparing their phenotype with the *ram1*Δ mutant in the future.

Fungal cell wall allows exchange of the compounds and signals between environment and fungal cells, or to build parasitical structures. Cell wall integrity (CWI) signal pathway is highly conserved in eukaryotic organisms, and is involved in stress tolerance and fungal pathogenicity (Hamel et al., 2012). We found that the *ram1*Δ sporidia were more tolerant to cell wall disrupting agent Congo red (CR), and CR treatment could induce a significantly higher up-regulation of several conserved CWI genes, especially Slt2 (MAPK) and Fks3 (glucan synthase), compared to that of wild-type sporidia. These results together demonstrate that Ram1 likely regulates the cell wall integrity in *S. scitamineum*.

In *S. scitamineum*, both *ras1*Δ and *ras2*Δ mutants were hypersensitive toward cell wall disrupt agents and cell membrane disrupt agents. In *C. neoformans*, intracellular cAMP level regulates the cell wall integrity pathway (Donlin et al., 2014), however, we found no obvious difference in terms of intracellular cAMP concentration of *S. scitamineum ras1*Δ or *ras2*Δ mutant compared to that of the wild-type strain. Sensitivity toward cell wall stressor Congo red was opposite in the *ras1*Δ or *ras2*Δ sporidia (hypersensitive) compared to that of *ram1*Δ mutant (tolerant). Both *ras1*Δ and *ras2*Δ were hypersensitive toward cell wall stressors, which supported the Ras involved in cell wall integrity pathway. But CFW dye on *ras2*Δ has almost no intensity but *ras1*Δ has strong intensity. From our unpublished data, three kinase genes involving in cell wall integrity pathway deletion mutants had slightly intensity by CFW dye, as the *ras2*Δ showed. We speculate the Ras1 and Ras2 involve in CWI pathway but have different compensation mechanism for cell wall composition. Overall, these results suggested that Ras may regulate *S. scitamineum* cell wall integrity through a signaling pathway independent or parallel of the cAMP/PKA pathway.

In summary, our study revealed a possible regulatory function of Ram1 by farnesylation of a-factor and/or Ras proteins, which are both critical for *S. scitamineum* mating/filamentation and/or stress tolerance.

## Data Availability

All datasets generated for this study are included in the manuscript and/or the Supplementary Files.

## Author Contributions

YD, BC, CC, and ZJ conceived and designed the experiments. SS, CC, EC, MY, and LL performed the experiments. YD, SS, CC, and ZJ analyzed the data and wrote the manuscript.

## Conflict of Interest Statement

The authors declare that the research was conducted in the absence of any commercial or financial relationships that could be construed as a potential conflict of interest.
