# Supplementary material for: The Farnesyltransferase β-Subunit Ram1 Regulates Sporisorium scitamineum Mating, Pathogenicity and Cell Wall Integrity
Source: Front Microbiol. 2019 May 8;10:976. doi: 10.3389/fmicb.2019.00976 (PMC6517510; doi:10.3389/fmicb.2019.00976)
Supplement: Supplementary file 1 [file Data_Sheet_1.pdf]

Supplementary Information

The farnesyltransferase β-subunit Ram1 regulates *Sporisorium scitamineum* mating, pathogenicity and cell wall integrity

Shuquan Sun, Yi Zhen Deng, Enping Cai, Meixin Yan, Lingyu Li, Baoshan Chen, Changqing Chang*, Zide Jiang*

* To whom correspondence should be addressed. Email: zdjiang@scau.edu.cn (Zide Jiang) or changcq@ scau.edu.cn (Changqing Chang)

This PDF file includes
Figure and Table Legends

Figure S1

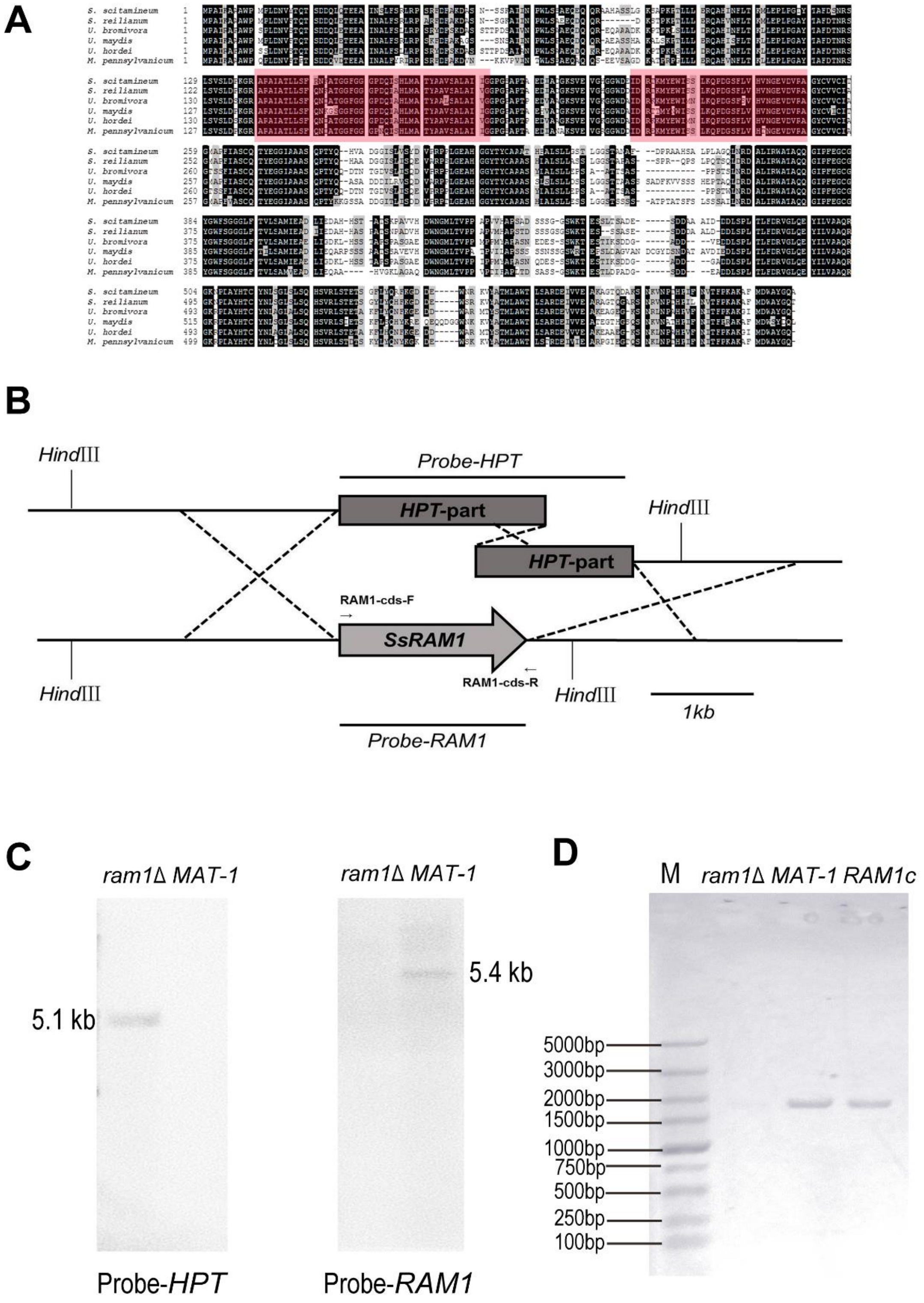

A
S. scitamineum
S. reilianum
U. bromivora
U. maydis
U. hordei
M. pennsylvanicum
B
HindIII
Probe-HPT
HPT-part
HPT-part
HindIII
RAM1-cds-F
SsRAM1
RAM1-cds-R
HindIII
HindIII
1kb
Probe-RAM1
C
ram1Δ MAT-1
5.1 kb
Probe-HPT
ram1Δ MAT-1
5.4 kb
Probe-RAM1
D
M ram1Δ MAT-1 RAM1c
5000bp
3000bp
2000bp
1500bp
1000bp
750bp
500bp
250bp
100bp

Figure S1 Construction and identification of *S. scitamineum ram1*Δ and *RAM1c*. (**A**) Amino acid sequences arrangement with *S. scitamineum* Ram1 protein and its orthologs in smut fungi by ClustalX2.0 and BioEdit. The black and grey shadow denoted identical and conserved residues respectively. The red boxes represent prenyltransferase and a squalene oxidase repeat (PF00432.16). (**B**) Schematic representation of generation of two partial-overlapped fragments with *HPT* resistant marker in replacement of *RAM1* gene locus. Solid lines represent sequences flanking *RAM1* gene, and dashed lines for the region where homologous recombination occurs. Localization of restriction enzyme *HindIII* and DNA fragments used as probes in Southern blot analysis in (C) were denoted. The scheme was drawn to scale with scale bar = 1 kb. (**C**) Southern blot to confirm *RAM1* gene deletion. Genomic DNA from wild-type (*MAT-1*) or selected transformant was digested with *Hind*Ш, and then probed with the *HPT* or *RAM1* fragment respectively. *HPT*-probing detected no band for the wild-type strain as expected, and a single band of 5.1 kb, diagnostic as *ram1*Δ mutant. *RAM1*-probing detected no band for *ram1*Δ mutant, confirming successful deletion of this gene, and a single band of 5.4 kb consistent with the calculated size resulted from specific gene replacement. (**D**) PCR verification of *ram1*Δ mutant and complimentary *RAM1c* strain, using the primer pair RAM1-cds-F/RAM1-cds-R, as denoted in (B).

Figure S2

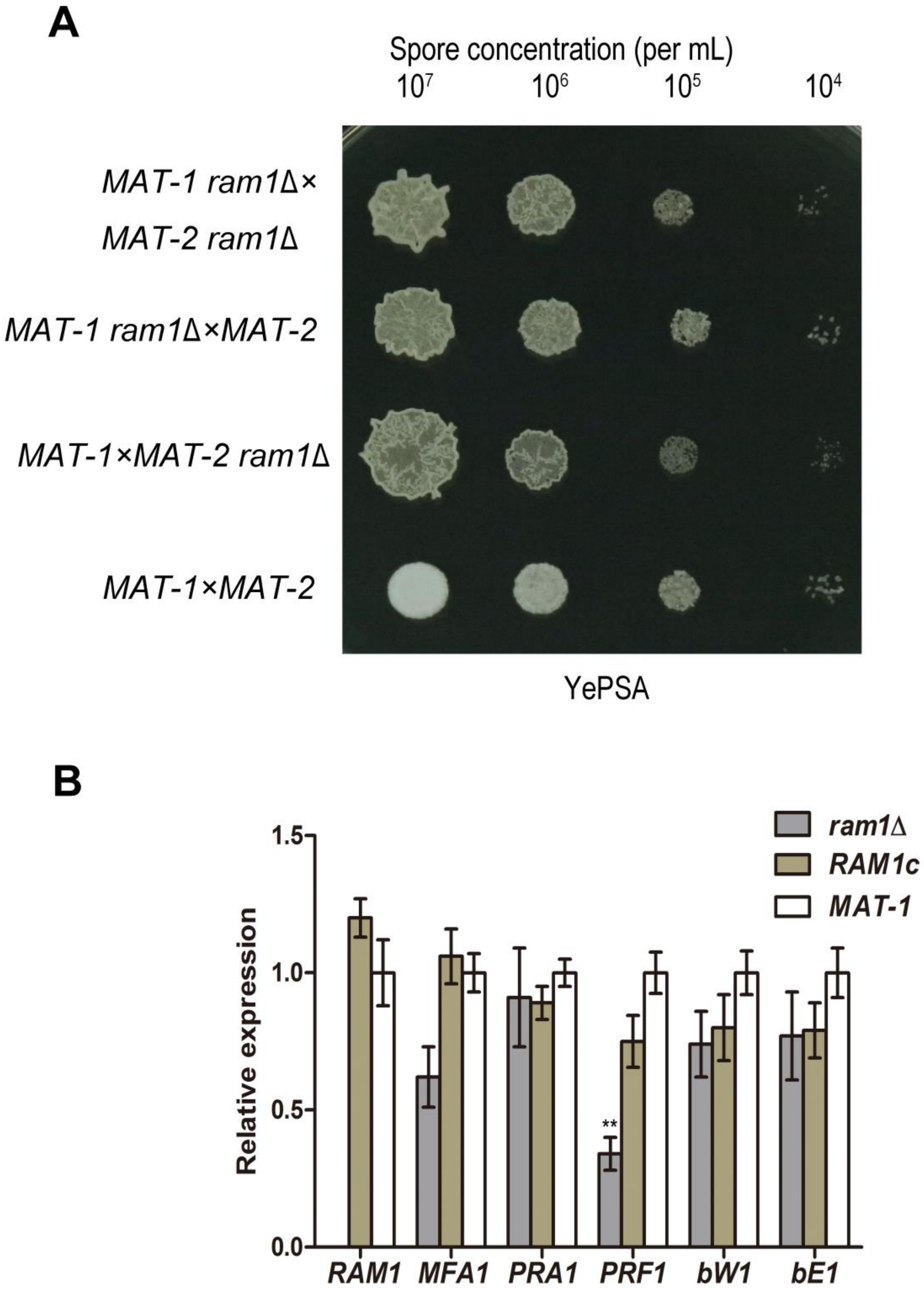
A
Spore concentration (per mL)
10^7 10^6 10^5 10^4
MAT-1 ram1Δ×
MAT-2 ram1Δ
MAT-1 ram1Δ×MAT-2
MAT-1×MAT-2 ram1Δ
MAT-1×MAT-2
YePSA
B
ram1Δ
RAM1c
MAT-1
Relative expression
1.5
1.0
0.5
0.0
**
RAM1 MFA1 PRA1 PRF1 bW1 bE1

Figure S2 *RAM1* regulates *S. scitamineum* mating/filamentation.

(**A**) A sporidial suspension (1 μL) of OD600=1.0, and the serial 10-fold dilutions of indicated strains, were spotted on YePSA plates and incubated at 28 ºC for 48 h, before examination and photographed. (**B**) qRT-PCR analysis of genes related to fungal mating and filamentation, in *ram1*Δ mutant compared to wild-type *MAT-1* or the complementation strain *RAM1c.* Sporidia were allowed to grow on YePSA plate for 36 h before total RNA extraction. Gene expression in the wild-type was set as 1 and the relative gene expression fold change in mutant or complementary strain was calculated with $2^{-\Delta\Delta Ct}$ method (Livak and Schmittgen 2001) using *ACTIN* as internal control. Statistical significance of the gene relative expressions was determined at $p < 0.01$ (**) using Student's t-test, and mean ± standard deviation was derived from three independent biological replicates.

Figure S3

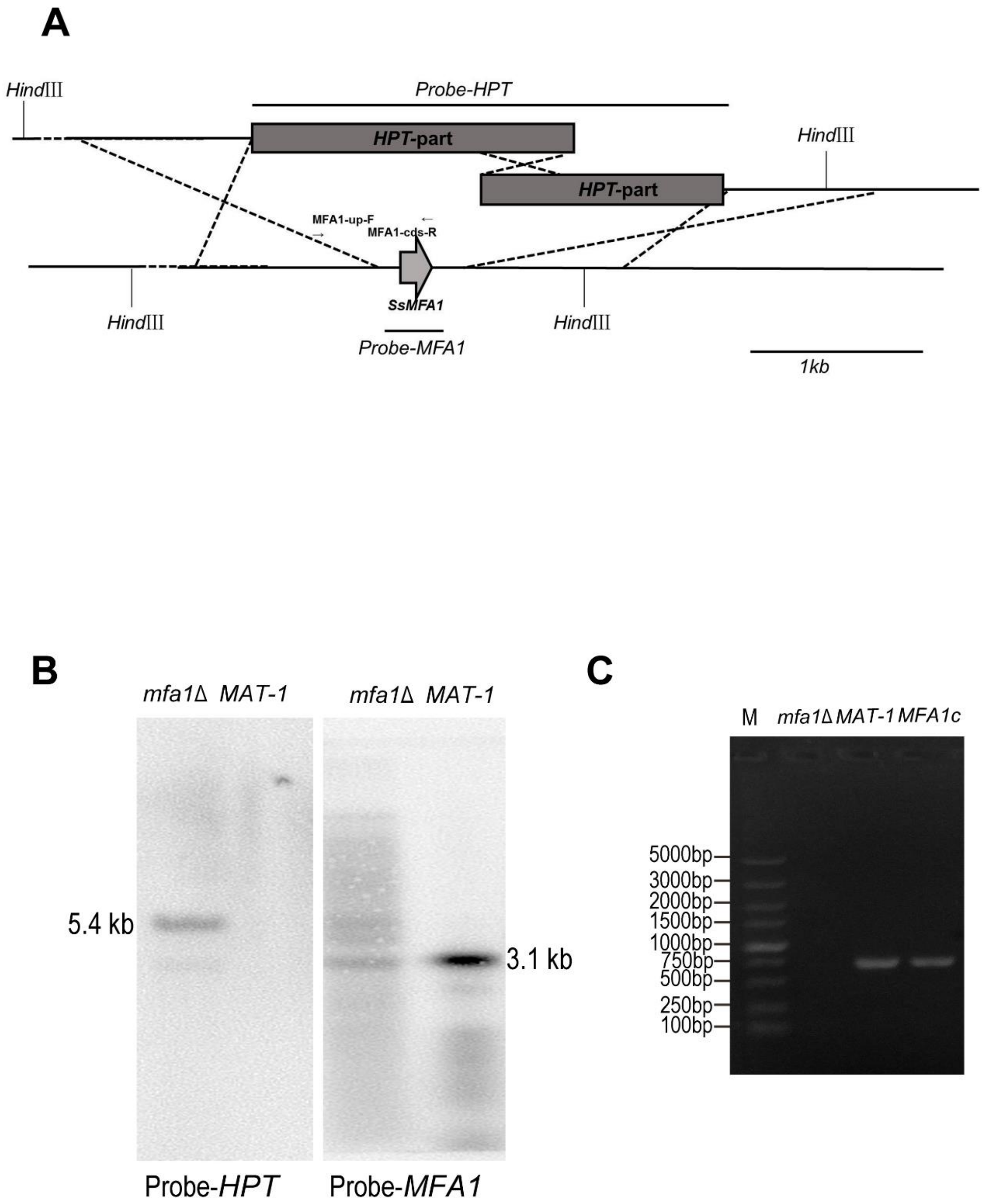
A
HindIII
Probe-HPT
HPT-part
HindIII
HPT-part
MFA1-up-F
MFA1-cds-R
HindIII
SsMFA1
HindIII
Probe-MFA1
1kb
B
mfa1Δ MAT-1
mfa1Δ MAT-1
5.4 kb
3.1 kb
Probe-HPT
Probe-MFA1
C
M mfa1Δ MAT-1 MFA1c
5000bp
3000bp
2000bp
1500bp
1000bp
750bp
500bp
250bp
100bp

Figure S3 Construction and identification of *S. scitamineum mfa1*Δ and *MFA1c* . (**A**) Schematic representation of generation of two partial-overlapped fragments with *HPT* resistant marker in replacement of *MFA1* gene locus. Solid lines represent sequences flanking *MFA1* gene, and dashed lines for the region where homologous recombination occurs. Localization of restriction enzyme *HindIII* and DNA fragments used as probes in Southern blot analysis in (B) were denoted. The scheme was drawn to scale with scale bar = 1 kb. (**B**) Southern blot to confirm *MFA1* gene deletion. Genomic DNA from wild-type (*MAT-1*) or selected transformant was digested with *Hind*Ш, and then probed with the *HPT* or *MFA1* fragment respectively. *HPT*-probing detected no band for the wild-type strain as expected, and a single band of 5.4 kb, diagnostic as *mfa1*Δ mutant. *MFA1*-probing detected no band for *mfa1*Δ mutant, confirming successful deletion of this gene, and a single band of 3.1 kb consistent with the calculated size resulted from specific gene replacement. (**C**) PCR verification of *mfa1*Δ mutant and complimentary *MFA1c* strain, using the primer pair MFA1-up-F /MFA1-cds-R, as denoted in (A).

Figure S4

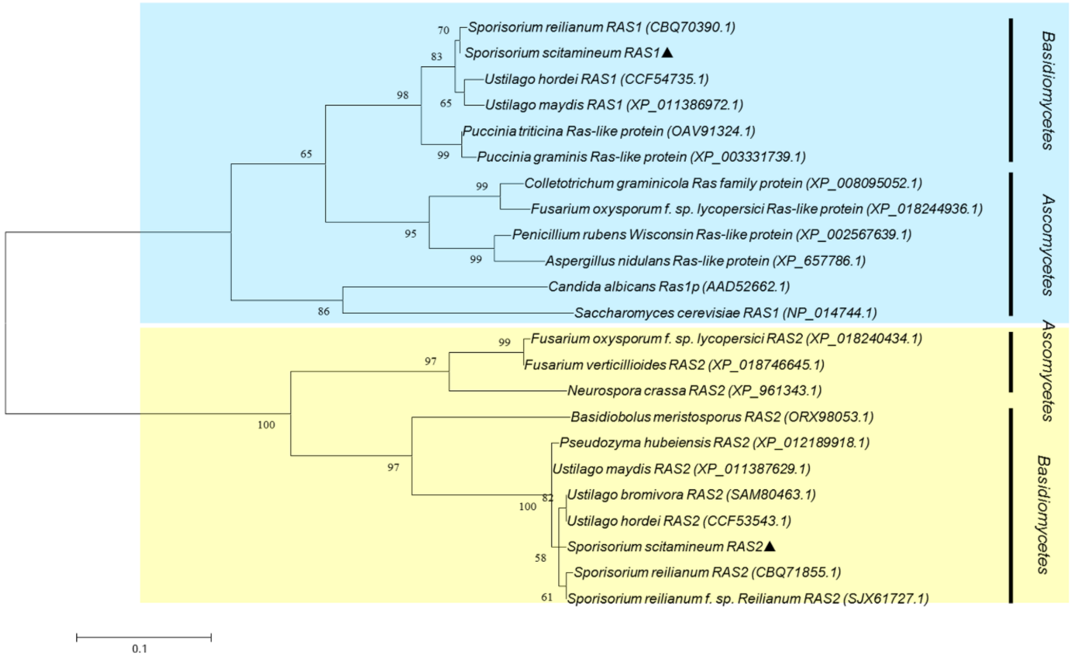

Sporisorium reilianum RAS1 (CBQ70390.1)
Sporisorium scitamineum RAS1▲
Ustilago hordei RAS1 (CCF54735.1)
Ustilago maydis RAS1 (XP_011386972.1)
Puccinia triticina Ras-like protein (OAV91324.1)
Puccinia graminis Ras-like protein (XP_003331739.1)
Colletotrichum graminicola Ras family protein (XP_008095052.1)
Fusarium oxysporum f. sp. lycopersici Ras-like protein (XP_018244936.1)
Penicillium rubens Wisconsin Ras-like protein (XP_002567639.1)
Aspergillus nidulans Ras-like protein (XP_657786.1)
Candida albicans Ras1p (AAD52662.1)
Saccharomyces cerevisiae RAS1 (NP_014744.1)
Fusarium oxysporum f. sp. lycopersici RAS2 (XP_018240434.1)
Fusarium verticillioides RAS2 (XP_018746645.1)
Neurospora crassa RAS2 (XP_961343.1)
Basidiobolus meristosporus RAS2 (ORX98053.1)
Pseudozyma hubeiensis RAS2 (XP_012189918.1)
Ustilago maydis RAS2 (XP_011387629.1)
Ustilago bromivora RAS2 (SAM80463.1)
Ustilago hordei RAS2 (CCF53543.1)
Sporisorium scitamineum RAS2▲
Sporisorium reilianum RAS2 (CBQ71855.1)
Sporisorium reilianum f. sp. Reilianum RAS2 (SJX61727.1)
Basidiomycetes
Ascomycetes
Ascomycetes
Basidiomycetes
70
83
98
65
65
99
99
95
99
86
99
97
100
97
82
100
58
61
0.1

Figure S4 Phylogenetic analysis with Ras1, Ras2 proteins and their orthologs. Phylogenetic analysis of fungal orthologous Ras1 proteins and Ras2 proteins. Amino acid sequences were aligned with ClustalX 2.0 with the following parameters: pairwise alignment parameters (gap opening = 10, gap extension = 0.1) and multiple alignment parameters (gap opening = 10, gap extension = 0.2, transition weight = 0.5, delay divergent sequences = 25%). The alignment was phylogenetically analyzed with maximum likelihood by MEGA 6.0, by using a Le-Gasquel amino acid replacement matrix with 1,000 bootstrap replications. *S. scitamineum* Ras1 and Ras2 proteins were denoted by solid triangles.

Figure S5

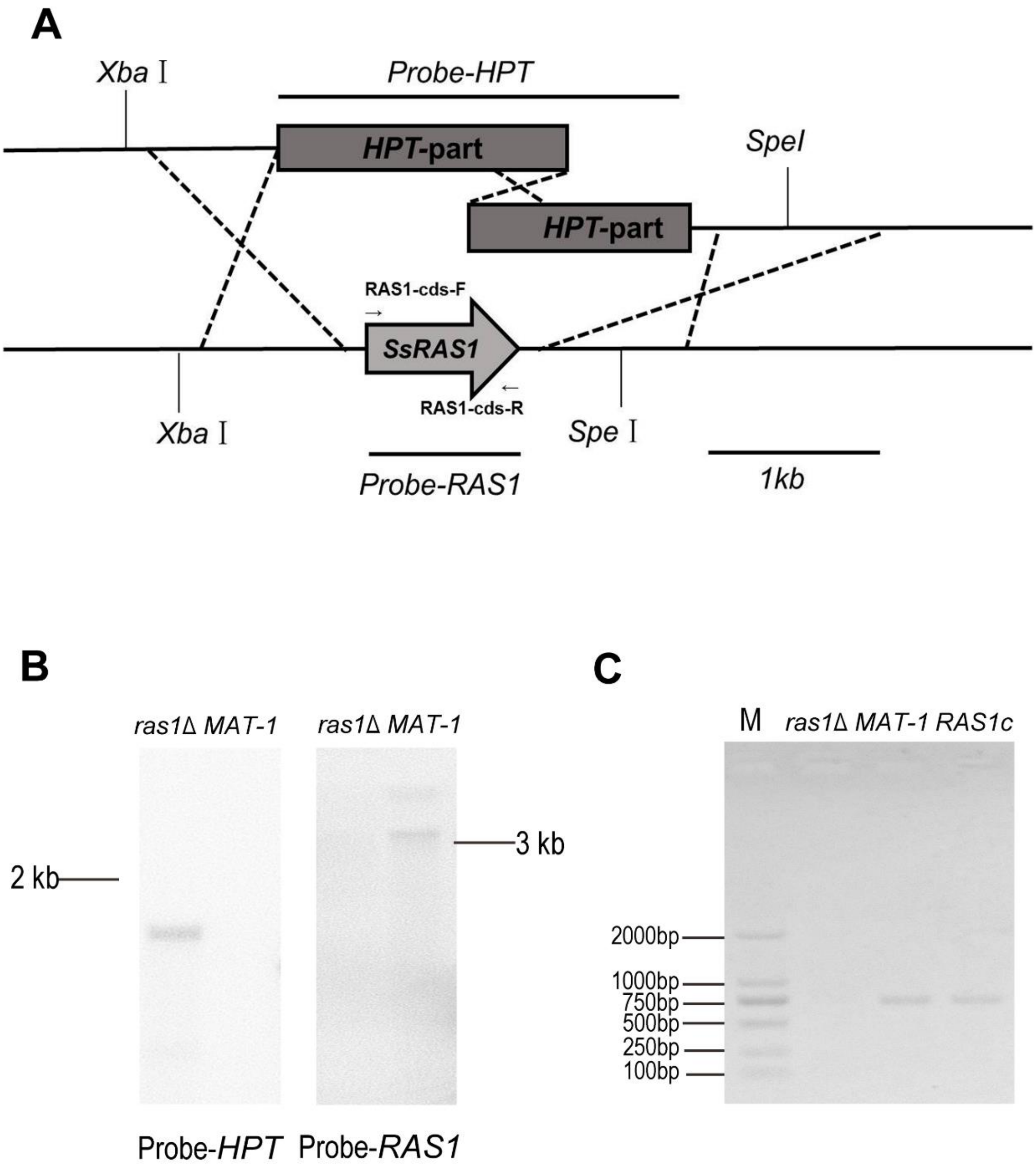
A
Xba I
Probe-HPT
HPT-part
SpeI
HPT-part
RAS1-cds-F
SsRAS1
RAS1-cds-R
Xba I
Spe I
Probe-RAS1
1kb
B
ras1Δ MAT-1
ras1Δ MAT-1
2 kb
3 kb
Probe-HPT
Probe-RAS1
C
M
ras1Δ MAT-1 RAS1c
2000bp
1000bp
750bp
500bp
250bp
100bp

Figure S5 Construction and identification of *S. scitamineum ras1*Δ and *RAS1c*.
(**A**) Schematic representation of generation of two partial-overlapped fragments with *HPT* resistant marker in replacement of *RAS1* gene locus. Solid lines represent sequences flanking *RAS1* gene, and dashed lines for the region where homologous recombination occurs. Localization of restriction enzyme *Xba*I and *Spe*I and DNA fragments used as probes in Southern blot analysis in (B) were denoted. The scheme was drawn to scale with scale bar = 1 kb. (**B**) Southern blot to confirm *RAS1* gene deletion. Genomic DNA from wild-type (*MAT-1*) or selected transformant was digested with *Xba*I and *Spe*I, and then probed with the *HPT* or *RAM1* fragment respectively. *HPT*-probing detected no band for the wild-type strain as expected, and a single band of 4.3 kb, diagnostic as *ras1*Δ mutant. *RAS1*-probing detected no band for *ras1*Δ mutant, confirming successful deletion of this gene, and a single band of 3.1 kb consistent with the calculated size resulted from specific gene replacement. (**C**) PCR verification of *ras1*Δ mutant and complimentary *RAS1c* strain, using the primer pair RAS1-cds-F/RAS1-cds-R, as denoted in (A).

Figure S6

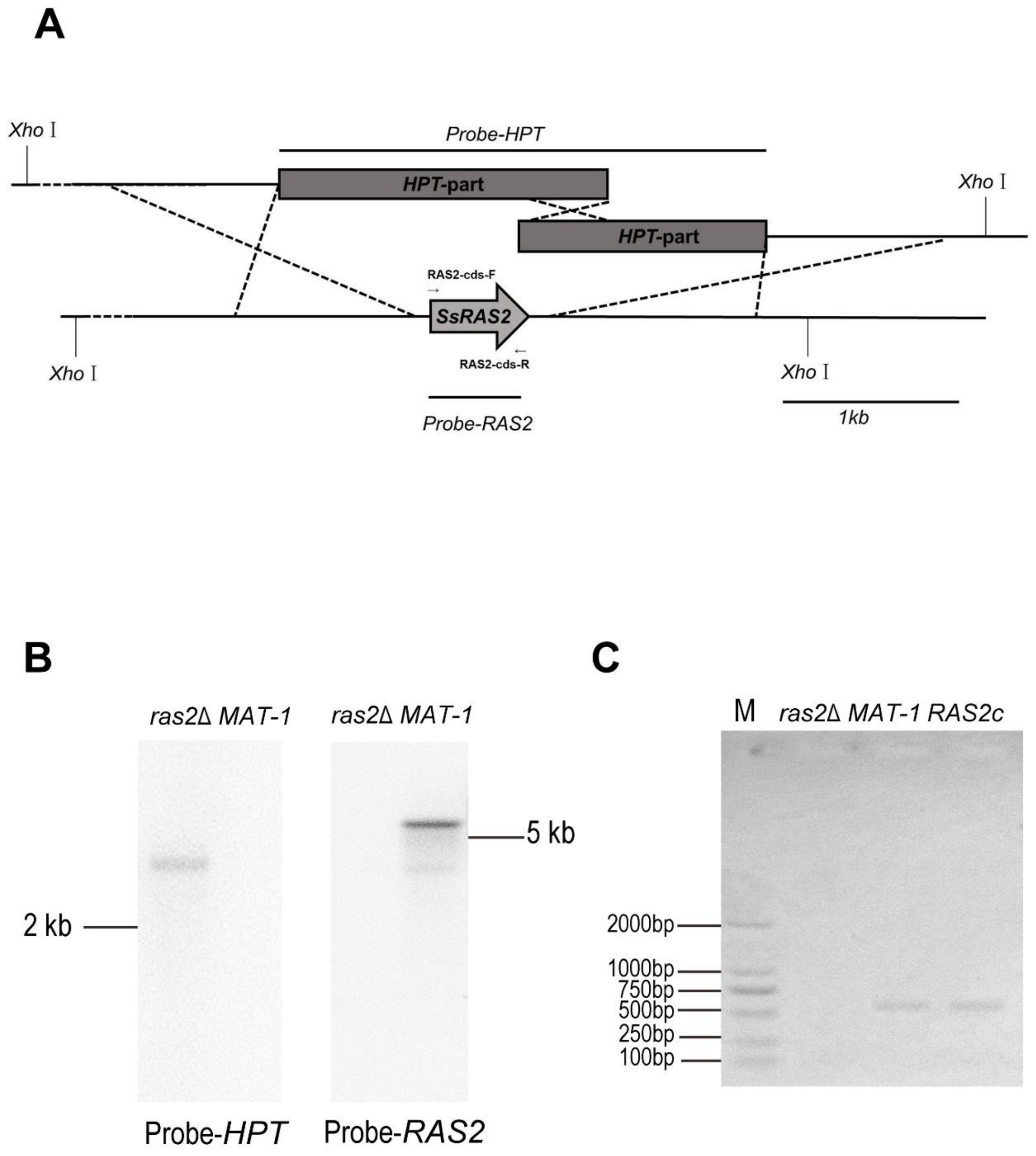
A
Xho I
Probe-HPT
HPT-part
HPT-part
Xho I
RAS2-cds-F
SsRAS2
RAS2-cds-R
Xho I
Xho I
Probe-RAS2
1kb
B
ras2Δ MAT-1
ras2Δ MAT-1
5 kb
2 kb
Probe-HPT
Probe-RAS2
C
M
ras2Δ MAT-1 RAS2c
2000bp
1000bp
750bp
500bp
250bp
100bp

Figure S6 Construction and identification of *S. scitamineum ras2*Δ and *RAS2c*.
(**A**) Schematic representation of generation of two partial-overlapped fragments with *HPT* resistant marker in replacement of *RAS2* gene locus. Solid lines represent sequences flanking *RAS2* gene, and dashed lines for the region where homologous recombination occurs. Localization of restriction enzyme *Xho*I and DNA fragments used as probes in Southern blot analysis in (B) were denoted. The scheme was drawn to scale with scale bar = 1 kb. (**B**) Southern blot to confirm *RAS2* gene deletion. Genomic DNA from wild-type (*MAT-1*) or selected transformant was digested with *Xho*I, and then probed with the *HPT* or *RAS2* fragment respectively. *HPT*-probing detected no band for the wild-type strain as expected, and a single band of 5.3 kb, diagnostic as *ras2* Δ mutant. *RAS2*-probing detected no band for *ram1*Δ mutant, confirming successful deletion of this gene, and a single band of 5.7 kb consistent with the calculated size resulted from specific gene replacement. (**C**) PCR verification of *ras2*Δ mutant and complimentary *RAS2c* strain, using the primer pair RAS2-cds-F/RAS2-cds-R, as denoted in (A).

Figure S7

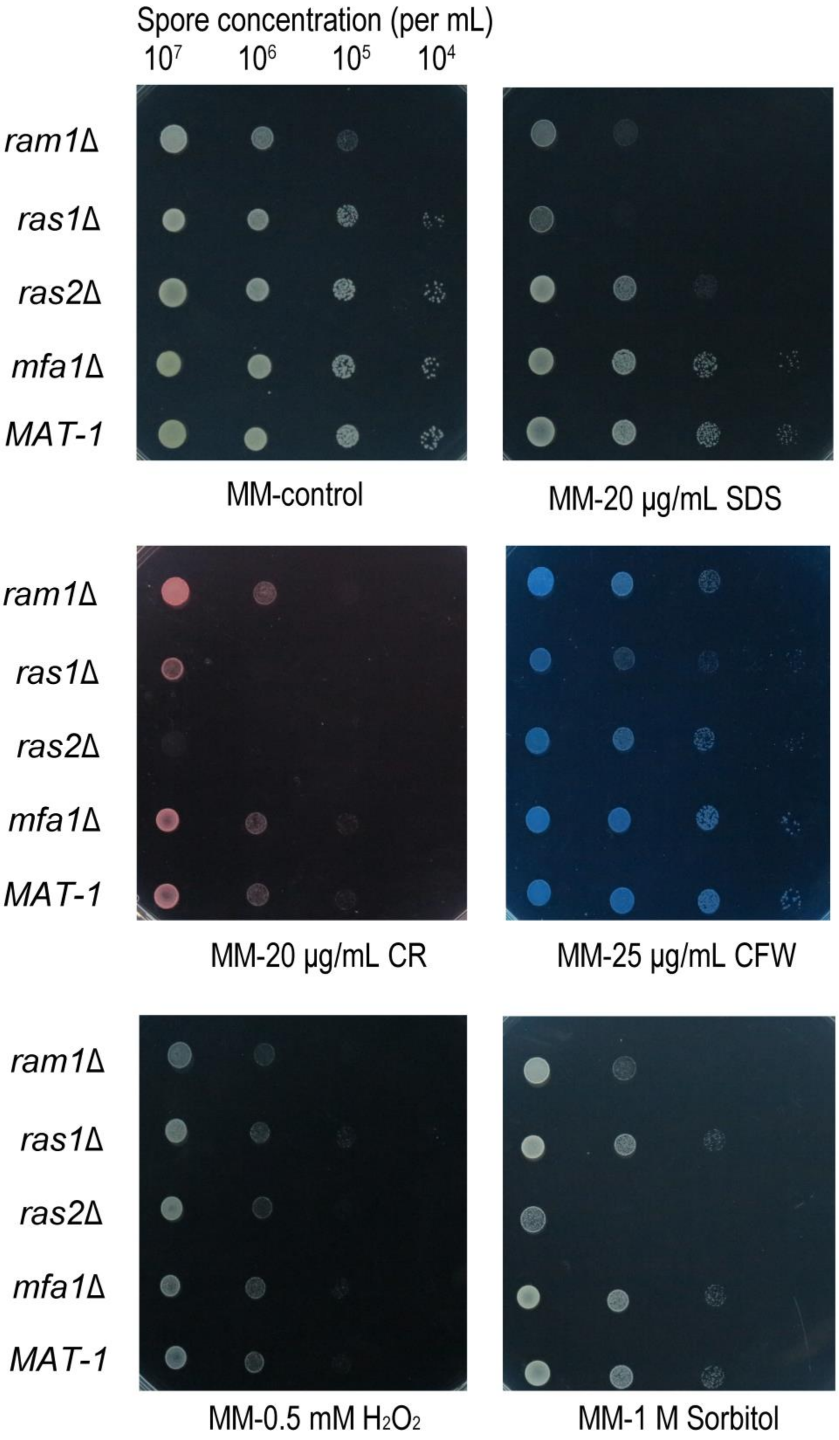
Spore concentration (per mL)
10^7 10^6 10^5 10^4
ram1Δ
ras1Δ
ras2Δ
mfa1Δ
MAT-1
MM-control
MM-20 μg/mL SDS
MM-20 μg/mL CR
MM-25 μg/mL CFW
MM-0.5 mM H2O2
MM-1 M Sorbitol

Figure S7 Stress tolerance assessment.

A sporidial suspension (1 μL) of OD600=1.0, and the serial 10-fold dilutions of *ram1*Δ, *ras1*Δ, *ras2*Δ, *mfa1*Δ mutants and wild-type *MAT-1* strains, were spotted on MM plates in the absence or presence of stress inducers, including 20 μg/mL Congo red, 25 μg/mL calcofluor white, 20 μg/mL SDS, 1 M Sorbitol and 0.5 mM H_2O_2, incubated in dark at 28 ºC for 48 h before examination.

Figure S8

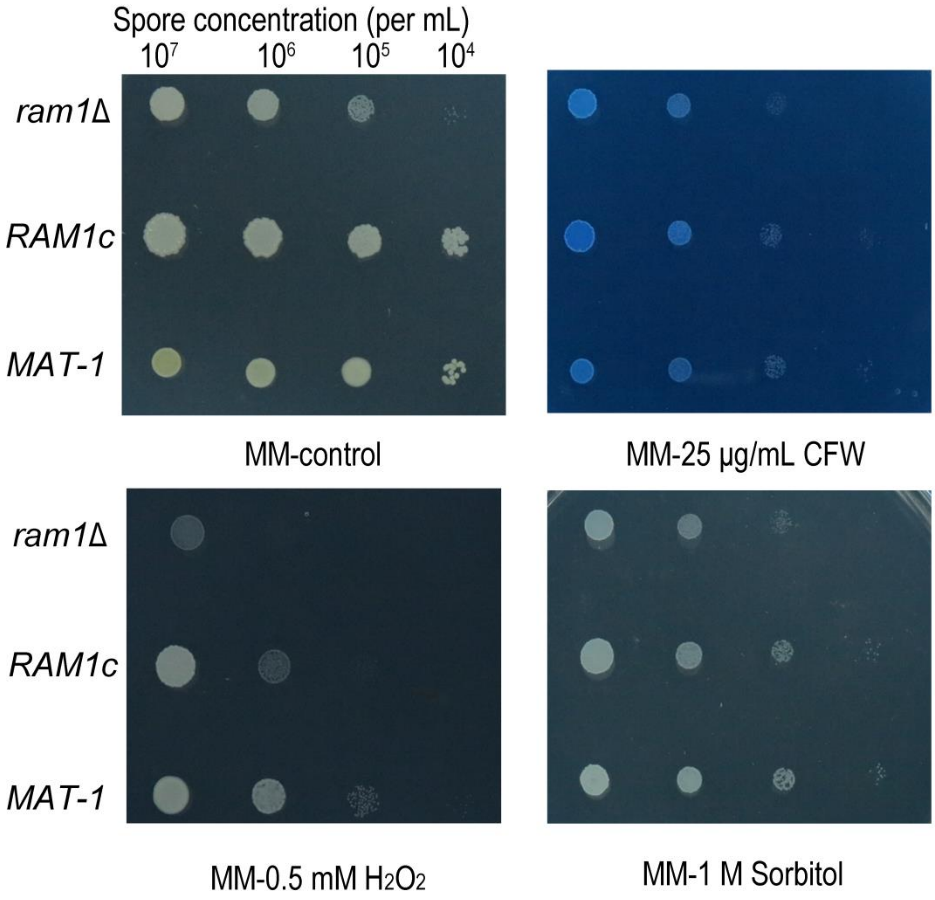
Spore concentration (per mL)
10^7 10^6 10^5 10^4
ram1Δ
RAM1c
MAT-1
MM-control
MM-25 μg/mL CFW
ram1Δ
RAM1c
MAT-1
MM-0.5 mM H2O2
MM-1 M Sorbitol

Figure S8 Assessment of stress tolerance with *ram1*Δ and *RAM1c*.

A sporidial suspension (1 µL) of OD600=1.0, and the serial 10-fold dilutions of *ram1*Δ, wild-type *MAT-1* and *RAM1c* strains, were spotted on MM plates in the absence or presence of stress inducers, including 25 µg/mL calcofluor white, 1 M Sorbitol and 0.5 mM H_2O_2, incubated in dark at 28 ºC for 48 h before examination.

Figure S9

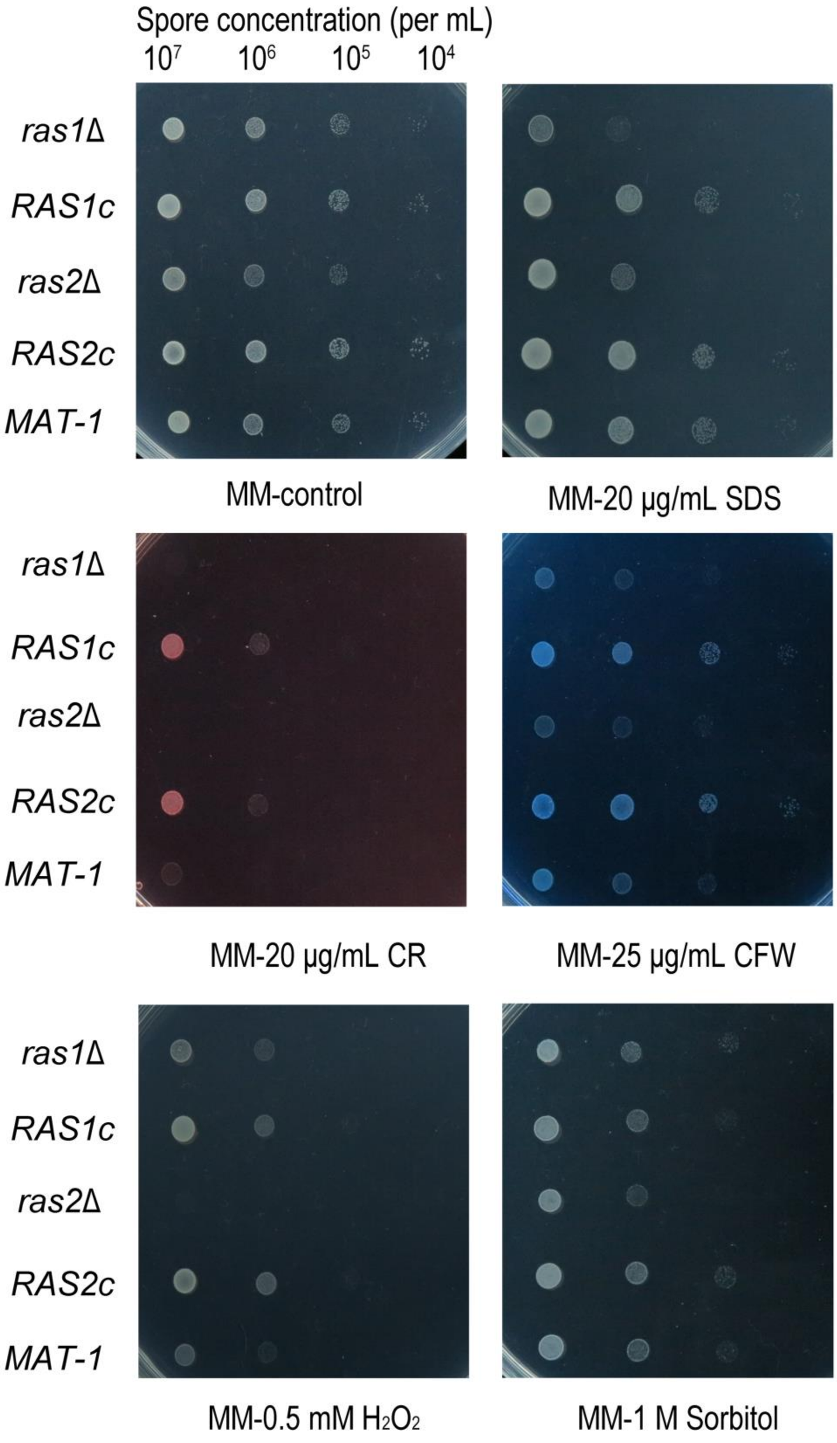
Spore concentration (per mL)
10^7 10^6 10^5 10^4
ras1Δ
RAS1c
ras2Δ
RAS2c
MAT-1
MM-control
MM-20 μg/mL SDS
MM-20 μg/mL CR
MM-25 μg/mL CFW
MM-0.5 mM H2O2
MM-1 M Sorbitol

Figure S9 Assessment of stress tolerance with *ras1*Δ, *ras2*Δ, *RAS1c* and *RAS2c* .
A sporidial suspension (1 μL) of OD600=1.0, and the serial 10-fold dilutions of *ras1*Δ, *ras2*Δ, *RAS1c, RAS2c* and wild-type *MAT-1* strains were spotted on MM plates in the absence or presence of stress inducers, including 20 μg/mL Congo red , 25 μg/mL calcofluor white, 20 μg/mL SDS , 1 M Sorbitol and 0.5 mM H_2O_2, incubated in dark at 28 ºC for 48 h before examination.

Table S1 List of target genes in this study

Gene name	Description	GenBank accession
RAM1	Beta subunit of farnesyltransferase	CP010926.1: 349030-350847
MFA1	Mating pheromone	CP010914.1: 857085-857204
RAS1	Small G-protein	CP010916.1: 737829-738566
RAS2	Small G-protein	CP010915.1: 596219-596794

Table S2 List of primers used for targeted gene deletion and complementary

Primer name	Primer sequences (5'to3')	Description
RAM1-LA-F	GTGAATGGTGCATGGCATCGG	
RAM1-LAHPT-R	GTCGTGACTGGGAAAACCCTGAGGCAAGAGGCAGCGTCAA	
RAM1-HPTRA-F	GGTCATAGCTGTTTCCTGTGTGAAGCGAATCTGTGCTTGCGGA	Deletion construction of *RAM1*
RAM1-RA-R	GCATCCACTGCGAATCGGC	
225-F	GCAAGACCTGCCTGAAACCG	
226-R	GGTCAAGACCAATGCGGAGC	
M13F	CAGGGTTTTCCCAGTCACGAC	
M13R	TCACACAGGAAACAGCTATGACC	
RAM1-cds-F	GCGTATTGTACAGTGGTGGGTGA	Deletion/ Complementary
RAM1-cds-R	CTCGATCGGTGGTCAGGCTTT	verification of *RAM1*
RAM1-com-F	TCCAAGCTCAAGCTAAGCTTTGAGTCGGCACTGCGTCAAA	Complementary construction of
RAM1-com-R	AGCAAGATCTAATCAAGCTTAGTATGGCGCGAAGGTGCTT	*RAM1*
RAS1-LA-F	CAGGCCATTGTGTGGGACTGA	Deletion construction of *RAS1*
RAS1-LAHPT-R	GTCGTGACTGGGAAAACCCTGGGTGATCCGGATGGCTGTTCT	
RAS1-HPTRA-F	GGTCATAGCTGTTTCCTGTGTGACATCCGTGCGCCATTTGACC	
RAS1-RA-R	CGCGTCTGTGTTGCGAAGTT	
RAS1-cds-F	ATGTCCAAGGCACAATTCTT	PCR verification/ Southern
RAS1-cds-R	GAGAACCACGCAACCGCTG	blotting probe
RAS1-com-F	TCCAAGCTCAAGCTAAGCTTCAAGTCACACGAGCCCACGAT	Complementary construction of
RAS1-com-R	CAGCAAGATCTAATCAAGCTGTTGCCTTCTTTGCTGCTGTGT	*RAS1*
RAS2-LA-F	ACATATCGTGTCGCCCTGCC	Deletion construction of *RAS2*
RAS2-LAHPT-R	GTCGTGACTGGGAAAACCCTGCACGCCTTCGATGGGTGCTA	
RAS2-HPTRA-F	GGTCATAGCTGTTTCCTGTGTGATGTCAGCAGCAGTGGTGTCG	

RAS2-RA-R	GACCGATGTACACCCGTTGTGA	
RAS2-cds-F	ATGAGTGGCAAAATGATGATCTAC	PCR verification/ Southern
RAS2-cds-R	AAGAATGTGGCAACGTGATTTCT	blotting probe
RAS2-com-F	TCCAAGCTCAAGCTAAGCTTCCCACGGTGAGCGAAAGGAT	Complementary construction of
RAS2-com-R	CAGCAAGATCTAATCAAGCTACCGTCGCACACTTCGCTT	*RAS2*
MFA1-LA-F	ACTTTGGCCACGGACTGGAC	Deletion construction of *MFA1*
MFA1-LAHPT-R	GTCGTGACTGGGAAAACCCTGTCCGTTGCGTGAATGGTTGC	
MFA1-HPTRA-F	GGTCATAGCTGTTTCCTGTGTGAACGGTGGATGTGATGACAGGG	
MFA1-RA-R	TCGCGGTGCTGACGAATGT	
MFA1-up-F	ATGCTTTCCATCTTTACCCA	PCR verification/ Southern
MFA1-cds-R	ACGGTGGATGTGATGACAGG	blotting probe
MFA1-com-F	TCCAAGCTCAAGCTAAGCTTGGAGCTGACTGGAGACGACG	Complementary construction of
MFA1-com-R	TCCAAGCTCAAGCTAAGCTTTACCGTCGCATGGGTTACGTC	*MFA1*
eGFP-F	AAAACACTCTTCCACCAAGCTTGTGAGCAAGGGCGAGGAGC	*ram1Δ/eGFP-RAM1*
eGFPRAM1-R	GCAGCTTTGATGGCTGGCATCTTGTACAGCTCGTCCATGCCGA	construction
eGFPRAM1-F	TCGGCATGGACGAGCTGTACAAGATGCCAGCCATCAAAGCTGC	
RAM1-R	CAGCAAGATCTAATCAAGCTCGCTTGACCATAAGCCCAA	

Table S3 List of selected genes primers used for qRT-PCR

GenBank accession	Gene name	Primer name	Primer sequences (5'to3')
CP010914.1	*MFA1*	MFA1-qPCR-F	ATGCTTTCCATCTTTACCCAGA
857085-857204		MFA1-qPCR-R	GTGCAGCTAGAGTAGCCAAG
AP014960.1	*GADPH*	GADPH-qPCR-F	CACGGCCACTGGAAGCA
22937203-22938694		GADPH-qPCR-R	TCCTCAGGGTTCCTGATGCC
CP010914.1	*bE1*	bE1-qPCR-F	TGAAAGTTCTCATGCAAGCC
793887-795381		bE1-qPCR-R	TGAGAGGTCGATTGAGGTTG
CP010914.1	*bW1*	bW1-qPCR-F	CACGTTGGATCTCGCTCGGT
795628-797775		bW1-qPCR-R	TCGGAAGGGAGGACGCAAAC
CP010914.1	*PRA1*	PRA1-qPCR-F	GCTCCAGTGCCGCAGTAAGT
859544-860833		PRA1-qPCR-R	GAATGTGGGCTTGCGTCGTC
CP010918.1	*PRF1*	PRF1-qPCR-F	GCCACCTCAGCCGTCTATCG
692187 -694352		PRF1-qPCR-R	ACTCGCAGTAGCCTTGCTCG
CP010926.1	*RAM1*	RAM1-qPCR-F	TGAATCGCGACGCCCTCATT
349030-350847		RAM1-qPCR-R	GAGGGACCGTGAGCATACCG
CP010916.1	*ACTIN*	ACTIN-qPCR-F	AAGTCGTACGAGCTTCCCGA
201251-202322		ACTIN-qPCR-R	TACCGGCGTACATGGTGGTA
CP010930.1	*WSC2*	WSC2-qPCR-F	GCCAACGCGAACCAGATGTG
484410-489113		WSC2-qPCR-R	TCTTCGCGCCCAATTGAGGT
CP010915.1	*MID2*	MID2-qPCR-F	CCTCGTCGTCCTCTGCTGAC
227643-229811		MID2-qPCR-R	GGCACCGCTGTCATCATCCT
CP010916.1	*ROM2*	ROM2-qPCR-F	CCGACACGCTTGGACCAGAT
575076-580106		ROM2-qPCR-R	ACGACGAGGTGACAGTGCTG
CP010933.1	*RHO1*	RHO1-qPCR-F	TCCCCATCATCCTCGTCGGT
199191-200160		RHO1-qPCR-R	GCTCAAACACTTCGCGCACA

CP010933.1	*PKC1*	PKC1-qPCR-F	CGGACGCTTGGGAGACATGT
226099-229827		PKC1-qPCR-R	GCGACTGTTGCATGTGAGGC
CP010915.1	*BCK1*	BCK1-qPCR-F	AGAACGCGTATGTGGCCCAA
661071-666884		BCK1-qPCR-R	CGCGCTCCAAACTCGTTCAC
CP010913.1	*SLT2*	SLT2-qPCR-F	TCAGGCGAGGATGATGCAGC
1237903-1239240		SLT2-qPCR-R	CCACTGGCATGCTCCGAGTT
CP010926.1	*RLM1*	RLM1-qPCR-F	GCTCCTTTGTCGGACCGGTT
265151-267019		RLM1-qPCR-R	CAACATGAACAGGCGGCGTT
CP010915.1	*FKS3*	FKS3-qPCR-F	AGAAGAAGCGCCTGGGACAC
577067-582421		FKS3-qPCR-R	TGGCGGCGTTCCAAACAATG
CP010918.1	*SMI1*	SMI1-qPCR-F	GTGCAATTCGAACAGGGCGG
765032-767347		SMI1-qPCR-R	TCGCTGTTCTACGCTGCTCC
CP010915.1	*SSK2*	SSK2-qPCR-F	TGGAGCAACAGCGATACGGG
322087-327779		SSK2-qPCR-R	ACTCTCGTCTCCAGCGGACT
CP010914.1	*HOG1*	HOG1-qPCR-R	TTCGTCCAGTCGTTGCCCAA
930343-931699		HOG1-qPCR-F	CGGGTTCGTCAGTAGGGTCG
